# A NADH Oxidase Nanozyme Restores Redox Homeostasis to Ameliorate Multi-Organ Aging and Ischemic Cardiomyopathy

**DOI:** 10.34133/research.0973

**Published:** 2025-10-28

**Authors:** Yu Chen, Yunsong Liang, Jie Shen, Yueyan Wang, Baoni Qiu, Honghao Hou, Xiaozhong Qiu

**Affiliations:** ^1^ The Fifth Affiliated Hospital, Southern Medical University, Guangzhou, Guangdong, 510900, People’s Republic of China.; School of Basic Medical Sciences, Southern Medical University, Guangzhou, Guangdong, 510515, People’s Republic of China.; ^3^ Guangdong Provincial Key Laboratory of Construction and Detection in Tissue Engineering, Guangzhou, Guangdong, 510515, People’s Republic of China.

## Abstract

Redox imbalance resulting from NAD^+^ [nicotinamide adenine dinucleotide (oxidized form)] depletion and NADH (reduced form of NAD^+^) accumulation is a conserved hallmark of both aging and myocardial infarction (MI), promoting cellular senescence and limiting the efficacy of regenerative therapies. Despite several NADH oxidase (NOX)-mimetic nanozymes having been reported, their therapeutic utility in aging and cardiovascular repair remains largely unexplored. Here, we present a vanadium-based nanozyme (MXene-TA) that mimics bacterial NOX activity, catalytically oxidizing NADH to restore NAD^+^ and directly fixing redox imbalance. In aged (24-month-old) mice, systemic MXene-TA administration restored NAD^+^/NADH homeostasis and reduced senescence markers (p16, p21, γH2AX, and SASP) in the heart, liver, and spleen, yet this effect was not observed in the lungs or kidneys, indicating organ-specific redox susceptibility. In a rat MI model, local injection of MXene-TA into the infarcted myocardium reprogrammed metabolism, activated NAD^+^-dependent pathways, attenuated oxidative damage in cardiomyocytes, decreased infarct area, and enhanced myocardial function. To further enhance stem cell retention and function, we embedded MXene-TA and adipose-derived stem cells (ADSCs) into a pH-responsive, conductive hydrogel that mimics cardiac mechanical and electrical properties. This platform extended ADSC survival beyond 4 weeks (versus 1 week in controls) and further improved cardiac repair. Together, these findings uncover the therapeutic potential of NOX-mimetic nanozymes in aging and ischemic heart disease and introduce a redox-regulating hydrogel system that addresses both oxidative stress and stem cell integration for effective myocardial repair.

## Introduction

Cardiovascular disease is one of the primary threats to global public health, among which myocardial infarction (MI) is a group of diseases marked by high morbidity and death rates [1]. MI impairs cardiac function, remodels cardiac structure, and ultimately leads to the progression of heart failure [2]. Among emerging regenerative strategies, ADSC-based therapies have shown promise [3], due to their low immunogenicity, ease of expansion, and multifaceted paracrine effects in promoting angiogenesis, immunomodulation, and cardiac repair [4–6]. Numerous preclinical studies have demonstrated consistent benefits of ADSC implantation in post-MI reconstruction of the left ventricle and heart failure [7,8]. However, therapeutic effects by ADSCs in MI remain limited in clinical settings. A key challenge is the harsh post-infarction microenvironment, which includes oxidative stress, metabolic dysfunction, and inflammation, any of which impair both the survival of resident cardiomyocytes and the transplanted stem cells [9,10].

Effective MI therapy thus requires a dual approach: protecting endogenous cardiomyocytes from oxidative injury and improving the viability and function of transplanted ADSCs. A key factor contributing to both cardiomyocyte damage and stem cell loss after MI is the marked metabolic disturbance during MI, especially the disruption of redox balance homeostasis [11]. Chronic ischemia causes metabolic reprogramming, resulting in a considerable buildup of NADH (reduced form of NAD^+^) and a reduction of NAD^+^ [nicotinamide adenine dinucleotide (oxidized form)] levels within cardiomyocytes and the nearby tissue environment [12–15]. This imbalance in the NAD^+^/NADH ratio impairs mitochondrial function, worsens oxidative stress, and reduces cellular energy metabolism. Numerous studies have shown that whether under in vitro hypoxia/reoxygenation conditions or in vivo ischemia/reperfusion injury, NAD^+^ levels consistently decrease: At the same time, NADH accumulates, contributing to both cardiomyocyte dysfunction and senescence of transplanted ADSCs [16,17]. Nicotinamide adenine dinucleotide (NAD) as an essential cofactor is widely distributed over the whole living cell [15]. Both its oxidized (NAD^+^) and reduced (NADH) states perform a critical function during metabolic processes in the electron transport chain, in which NAD^+^ serves as an acceptor of electrons in cellular redox processes. These reactions are vital for extracting energy from glucose and fatty acids, which are necessary for maintaining essential cellular functions [18]. NAD^+^ affects many vital cellular processes, such as DNA damage repair, remodeling chromatin, cellular senescence, and the function of immune cells. [19]. While NAD^+^ precursor supplementation ([e.g., nicotinamide mononucleotide (NMN) and nicotinamide riboside (NR)] can partially elevate NAD^+^ levels, their limited bioavailability, high cost, and risk of NADH overload or tissue-specific toxicity remain substantial challenges for clinical apply [20]. These limitations underscore the need for novel approaches that can directly and efficiently modulate NAD^+^/NADH metabolism within the infarcted microenvironment to support cardiomyocyte protection and stem cell engraftment simultaneously.

New advances in the field of nanozyme technology have led to the formulation of over 30 types of NADH oxidase (NOX)-mimicking nanozymes [21]. These nanozymes typically demonstrate strong catalytic activity for NADH oxidation in vitro and have been examined in contexts such as tumor metabolism or redox environment regulation [22–25]. However, their efficacy in modulating NAD^+^ metabolism in vivo, particularly in organ-level aging or ischemic heart repair, remains largely unverified. In the context of MI, where both endogenous cardiomyocytes and transplanted ADSCs face redox stress, it remains unclear whether NOX-like nanozymes can restore the NAD^+^/NADH balance, reduce oxidative damage, and improve therapeutic outcomes. Furthermore, most existing nanozymes have limited catalytic specificity and biocompatibility, and lack mechanisms for microenvironmental responsiveness in infarcted tissues [21].

To address these challenges, we screened a series of transition metal-based MXene materials, known for their large surface area, high electrical conductivity, and catalytic versatility [24,26–31]. We first constructed a NOX-mimetic nanozyme by modifying vanadium-based VOx-V_2_C MXene with tannic acid, resulting in a biocompatible and catalytically active material termed MXene-TA. We further evaluated its systemic anti-aging potential in vivo. In naturally aging mice, 6 weeks of daily injections of MXene-TA into the abdominal cavity significantly elevated NAD^+^ levels and NAD^+^/NADH ratios in the heart, liver, and spleen, along with reduced NADH accumulation and decreased expression of senescence markers (p16, p21, γH2AX). No histological damage was observed in major organs, confirming the biosafety of MXene-TA. To evaluate its effectiveness in cardiac disease, we next tested MXene-TA in vitro and a rat model of MI. In vitro, MXene-TA increased the antioxidant capacity of primary cardiomyocytes and reduced senescence in ADSCs, indicating its dual benefit in supporting both native and engrafted cells. Direct delivery of MXene-TA into the infarcted myocardium triggered metabolic reprogramming, as evidenced by higher levels of NAD^+^, adenosine triphosphate (ATP), nicotinamide, and glutamine, along with the activation of oxidative phosphorylation, NAD^+^ biosynthesis, and pathways related to longevity. This resulted in reduced infarct area, improved myocardial survival, and lower early mortality. To improve ADSC viability and enable localized delivery, we encapsulated both MXene-TA and ADSCs into a pH-responsive, conductive hydrogel system made of polyvinyl alcohol (PVA), oxidized sodium alginate (OSA), and borate ester linkages. This hydrogel mimicked the biomechanical and electrical properties of native myocardium, allowing for the controlled release of MXene-TA in the acidic infarct microenvironment. Upon implantation into the infarct zone, the MXene-TA-loaded hydrogel significantly extended the survival of transplanted ADSCs and supported their functions, resulting in a continuous improvement in cardiac function. Overall, this study shows that MXene-TA functions as a versatile NOX-like nanozyme with systemic anti-aging properties and redox reprogramming potential. When incorporated into a bioresponsive hydrogel platform, it effectively tackles both oxidative damage and stem cell engraftment issues, offering a powerful therapeutic strategy for MI repair*.*

## Results

### MXene-TA possesses well-characterized NOX activity

**Fig. 1.**
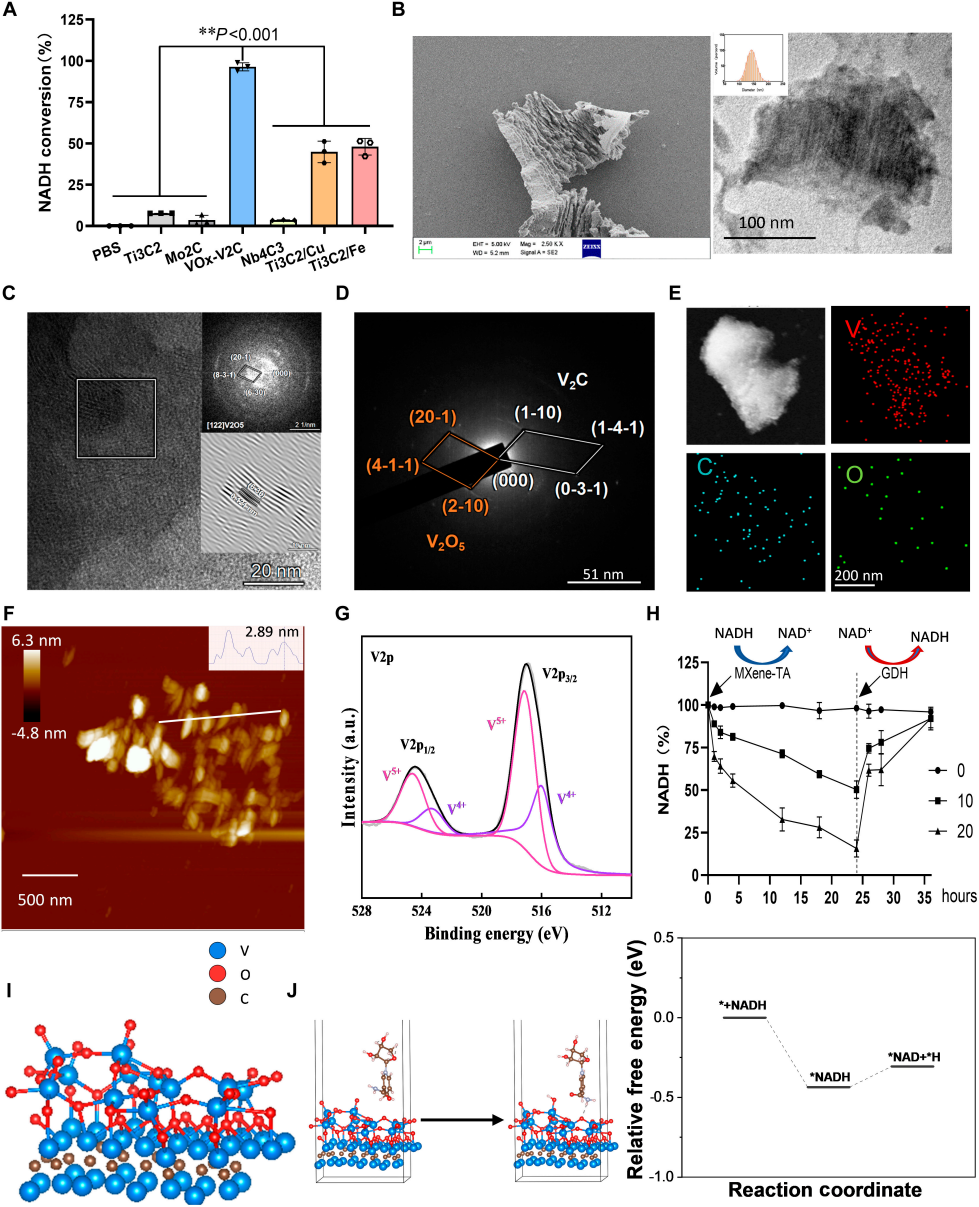
Characterization of MXene-TA materials. (A) The catalytic activity of MXene material (200 μg/ml) was evaluated by monitoring the change in the absorbance of the substrate in the WST-8 assay. (B) Characterization of the physicochemical properties of MXene-TA by SEM, TEM, and DLS. (C) High-resolution TEM (HRTEM) images of MXene-TA. (D) Selected-area electron diffraction (SAED) pattern. (E) Scanning transmission electron microscopy–energy-dispersive x-ray spectroscopy (STEM EDX) elemental mapping images of MXene-TA. (F) AFM images and height distribution. (G) Powder XPS patterns. (H) The proportion of NADH converted by MXene-TA into NAD^+^ was detected using a colorimetric assay. (I) Crystal model of VOx-V_2_C MXene. (J) Free energy profiles of NADH conversion on VOx-V_2_C MXene surface. Quantitative data were expressed as the mean ± SD of at least 3 independent experiments. **P < 0.01.

By changing the type of transition metal during the synthesis process, 6 MXene catalytic materials were prepared: Ti_3_C_2_, Mo_2_C, VOx-V_2_C, Nb_4_C_3_, Ti_3_C_2_/Gu, and Ti_3_C_2_/Fe. The NADH catalytic activity was detected by the reaction of the colorimetric substrate WST-8. As shown in Fig. 1A, VOx-V_2_C exhibited a high catalytic activity for NADH, and the conversion of NADH could reach more than 80% after 2 h under the catalytic action of VOx-V_2_C. The conversion of Ti_3_C_2_/Gu and Ti_3_C_2_/Fe was generally effective (about 50%), while in the presence of Ti_3_C_2_, Mo_2_C, and Nb_4_C_3_, the conversion rate was only about 10%. Considering that vanadium ions can regulate glycolipid metabolism and immunomodulation at certain doses, but excessive intake may trigger a toxic response, we examined the cytotoxicity of VOx-V_2_C MXene, and the results showed significant toxicity to cardiomyocytes when the concentration of VOx-V_2_C MXene reached 12 μg/ml (Fig. S1A). To reduce the cytotoxicity of VOx-V_2_C MXene, we introduced tannins, which have multiple phenolic hydroxyl groups that can form stable complexes with vanadium ions, a process known as chelation (Fig. S1B). This significantly reduced the cytotoxicity of vanadium ions (Fig. S1B) without lowering the NADH oxidation activity of VOx-V_2_C MXene (Fig. S1C).

To confirm the successful synthesis of VOx-V_2_C MXene, we performed high-resolution scanning transmission electron microscopy (STEM) analysis. Scanning electron microscopy (SEM) showed that MXene-TA displayed a lamellar structure with distinct layer spacing, indicating the material’s high specific surface area. The size of MXene-TA was about 150 nm, as detected by transmission electron microscopy and dynamic light scattering (DLS) after ultrasonically crushing and resuspending (Fig. 1B). As shown in Fig. 1C, the measured interplanar spacing of 0.121 nm corresponds to the (6–30) plane of V_2_O_5_, and the corresponding fast Fourier transform (FFT) pattern (inset) reveals the coexistence of crystalline and amorphous regions with lattice defects and distortions. The selected-area electron diffraction (SAED) pattern (Fig. 1D) displays concentric rings with distinct diffraction spots corresponding to V_2_C and V_2_O_5_ phases, while the presence of diffuse rings without diffraction spots further confirms the amorphous domains within VOx-V_2_C MXene. This unique crystalline–amorphous interlaced structure is expected to buffer volume changes and enhance structural stability. In addition, elemental mapping (Fig. 1E) demonstrates the homogeneous distribution of V, O, and C elements across the VOx-V_2_C MXene. Figure 1F shows the atomic force microscopy (AFM) image of VOx-V_2_C MXene, and the average thickness of the single-layer MXene sheet is approximately 2.89 nm. The x-ray photoelectron spectroscopy (XPS) analyzed the elemental composition and valence states of MXene-TA. High-resolution V2p spectra (Fig. 1G) reveal that the vanadium metal in MXene-TA mainly exists in the pentavalent and tetravalent states. The high valence state of the vanadium metal indicates good oxidation ability. Furthermore, the coexistence of multiple oxidation states, particularly the redox-active V^5+^, coupled with the material’s high specific surface area, synergistically contributes to NADH oxidation activity. These findings suggest that MXene-TA has promise as a biocompatible nanocatalyst.

To evaluate the NADH catalytic properties of MXene-TA, we reacted MXene-TA at a range of concentrations between 0 and 100 μg/ml with an NADH solution and measured the NADH clearance rate for 72 h. Figure S1D to F shows that as the concentration of MXene-TA and the duration of the reaction increase, the NADH clearance rate progressively rises. To further assess MXene-TA’s ability to catalyze the conversion of NADH to NAD^+^, we added MXene-TA to the NADH solution. Then, we introduced glucose dehydrogenase (GDH), which catalyzes the conversion of NAD^+^ to NADH. Figure 1H indicates that after the addition of MXene-TA, NADH was converted to NAD, and the subsequent addition of GDH fully converted NAD^+^ back to NADH. In biological systems, the NOX enzyme catalyzes the oxidation–dehydrogenation reaction of NADH, producing hydrogen peroxide or water. Considering the cytotoxic effects of excessive hydrogen peroxide, we assessed the production of hydrogen peroxide during the NADH catalytic process using MXene-TA. Figure S1G demonstrates that MXene-TA did not produce hydrogen peroxide during the NADH catalytic process. To understand the origin of the NOX-like catalytic activity of VOx-V_2_C MXene, we calculated the crystal model of VOx-V_2_C MXene using first-principles calculations (Fig. 1I) and density functional theory calculations to explore its catalytic behavior and calculate the free energy changes along the optimal reaction path. As shown in Fig. 1J, the NADH molecule can easily adsorb onto VOx-V_2_C MXene, with adsorption energies of −0.43 eV, indicating that VOx-V_2_C MXene has the highest ability to capture NADH molecules. Additionally, VOx-V_2_C MXene has a lower energy barrier (0.1 eV) in the key step of dehydrogenation, which demonstrates its excellent NOX-like activity. These results indicated that MXene-TA functions as a water-producing NOX-like nanoenzyme, exhibiting high catalytic efficiency, stability, and notable biocompatibility.

### MXene-TA regulates NAD^+^/NADH balance to alleviate multi-organ aging

Considerable research has demonstrated that NAD^+^ supplementation or its precursor substances,  like nicotinamide (NAM), nicotinic acid (NA), and NMN, can potently elevate the levels of NAD^+^ in tissues, thereby significantly slowing down the aging process in several organs and skin [32,33]. However, most existing studies focus on the anti-aging effects of NAD^+^ supplements, and there are still limited studies on regulating intracellular redox status by enhancing NADH oxidation, particularly the use of nanozymes with NADH oxidation function to delay organ aging. Based on this, in the present study, 24-month-old naturally senescent C57BL/6 mice were selected and randomly assigned to groups, and then treated with daily intraperitoneal injections of phosphate-buffered saline (PBS) or MXene-TA (8 μg/ml) for 6 consecutive weeks to assess its effects on organ metabolic status and senescence markers. In determining its potential biotoxicity, hematoxylin and eosin (H&E) staining (Fig. S2A) revealed that no significant histological injury was observed in the organs of MXene-TA-treated mice, suggesting that MXene-TA exhibits good biocompatibility at this dose and treatment cycle.

**Fig. 2.**
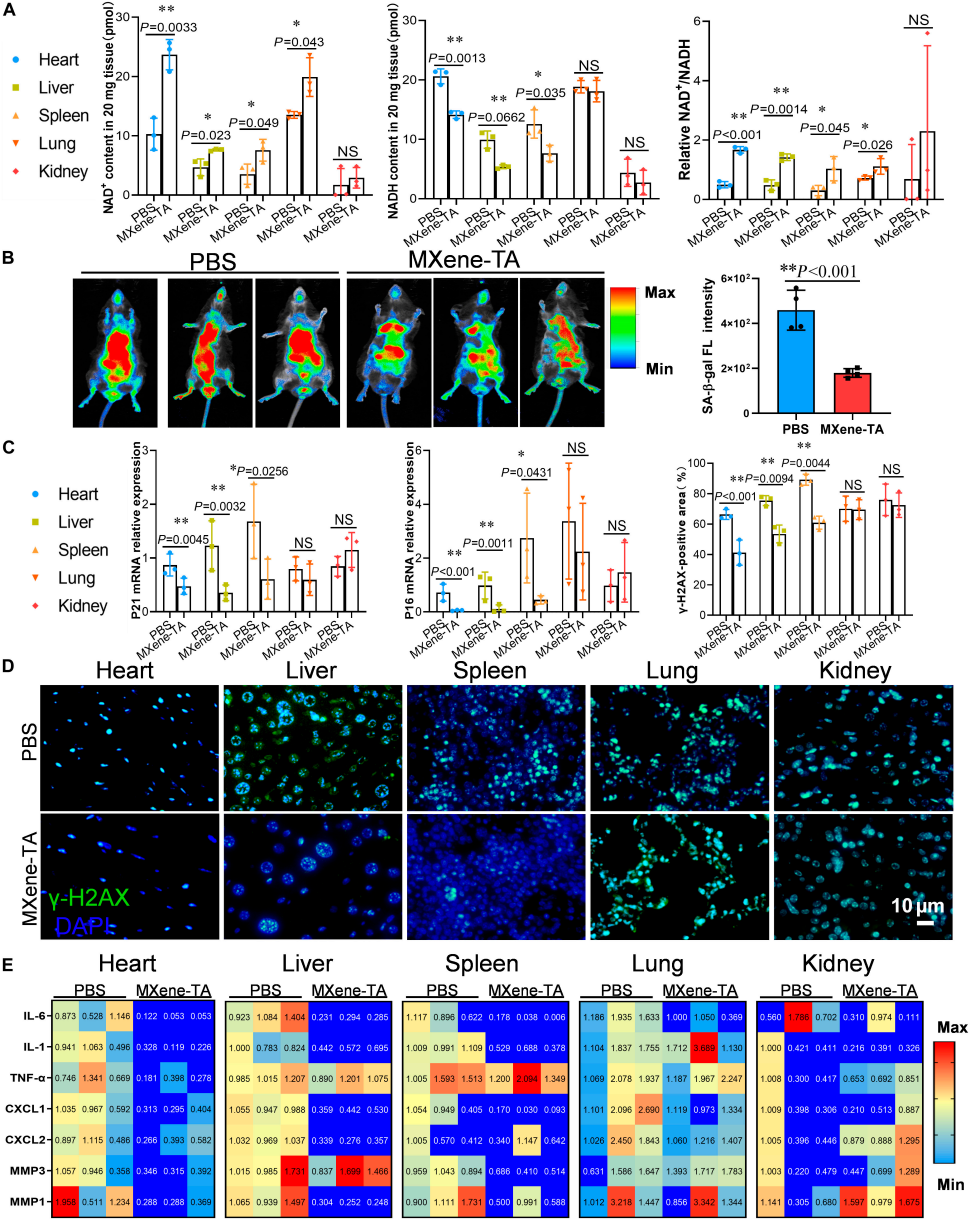
MXene-TA alleviates multiple organ aging phenotypes in naturally aged model mice. (A) Quantification of NAD^+^ levels, NADH levels, and NAD^+^/NADH ratios in the heart, liver, spleen, lung, and kidney (*n* = 3 rats per group). (B) In vivo SA-β-gal bioluminescence imaging showing reduced systemic senescence signals in MXene-TA-treated mice (*n* = 4). (C) mRNA expression levels of senescence markers p16 and p21 in major organs (*n* = 3 rats per group). (D) Immunofluorescence staining and quantification of DNA damage marker γH2AX in various organs. Scale bar, 10 μm; *n* = 3 rats per group. (E) mRNA levels of SASP-related cytokines [e.g., IL-6, tumor necrosis factor-α (TNF-α), and matrix metalloproteinase 3 (MMP3)] were reduced in the same organs (*n* = 3 rats per group). Quantitative data were expressed as the mean ± SD. *P < 0.05; **P < 0.01; NS, not significant.

Subsequently, we examined the content of NAD^+^ and NADH as well as NAD^+^/NADH ratio in 5 major organs (Fig. 2A). The results showed that MXene-TA significantly elevated the NAD^+^ level and NAD^+^/NADH ratio in heart, liver, and spleen, accompanied by a remarkably declined level in NADH content, demonstrating that it may improve the cellular metabolic state by enhancing the NADH oxidation process, thereby delaying aging. In lung tissues, NAD^+^ levels and NAD^+^/NADH ratios were also elevated. Still, there was no significant change in NADH content, which was considered to be possibly related to the lower production of NADH itself in the hyperoxic environment of lung tissues [34,35]. In the kidney, there were no significant changes in NAD^+^ and NADH levels or their ratios, likely due to the large difference in oxygen supply between the renal cortex and medulla, which resulted in a nonsignificant change in overall NAD^+^ metabolism status [36]. To further assess the degree of senescence, in vivo imaging confirmed the effect of MXene-TA on the overall senescence status, and the experimental results showed a decrease in senescence-associated β-galactosidase (SA-β-gal) activity in the MXene-TA-treated group of mice (Fig. 2B). In contrast, the method was unable to distinguish organ-specific changes.

To clarify the target organs for senescence relief, we measured the level of mRNA expression for the cell cycle inhibitory proteins p16 and p21 (Fig. 2C). The experimental results indicate that the p16 and p21 expression levels in the heart, liver, and spleen of the MXene-TA-treated group were significantly down-regulated, while no obvious alterations were observed in the lungs and kidneys. Additionally, senescent cells were usually accompanied by up-regulation of the DNA damage marker γ-H2AX and aberrant expression of senescence-associated secretory phenotype (SASP) factors. We further detected the γ-H2AX protein content and SASP-related mRNA levels (Fig. 2D and E), and the results showed that MXene-TA treatment significantly decreased the γ-H2AX levels and SASP gene expression in the heart, liver, and spleen. At the same time, the changes in the lungs and kidneys were not obvious. In summary, the MXene-TA can regulate the balance of NAD^+^/NADH metabolism to alleviate the aging state of multiple organs, especially in organs where the NADH to NAD^+^ ratio is severely imbalanced, such as the heart, liver, and spleen.

### MXene-TA nanozymes decreased oxidative damage in cardiomyocytes

**Fig. 3.**
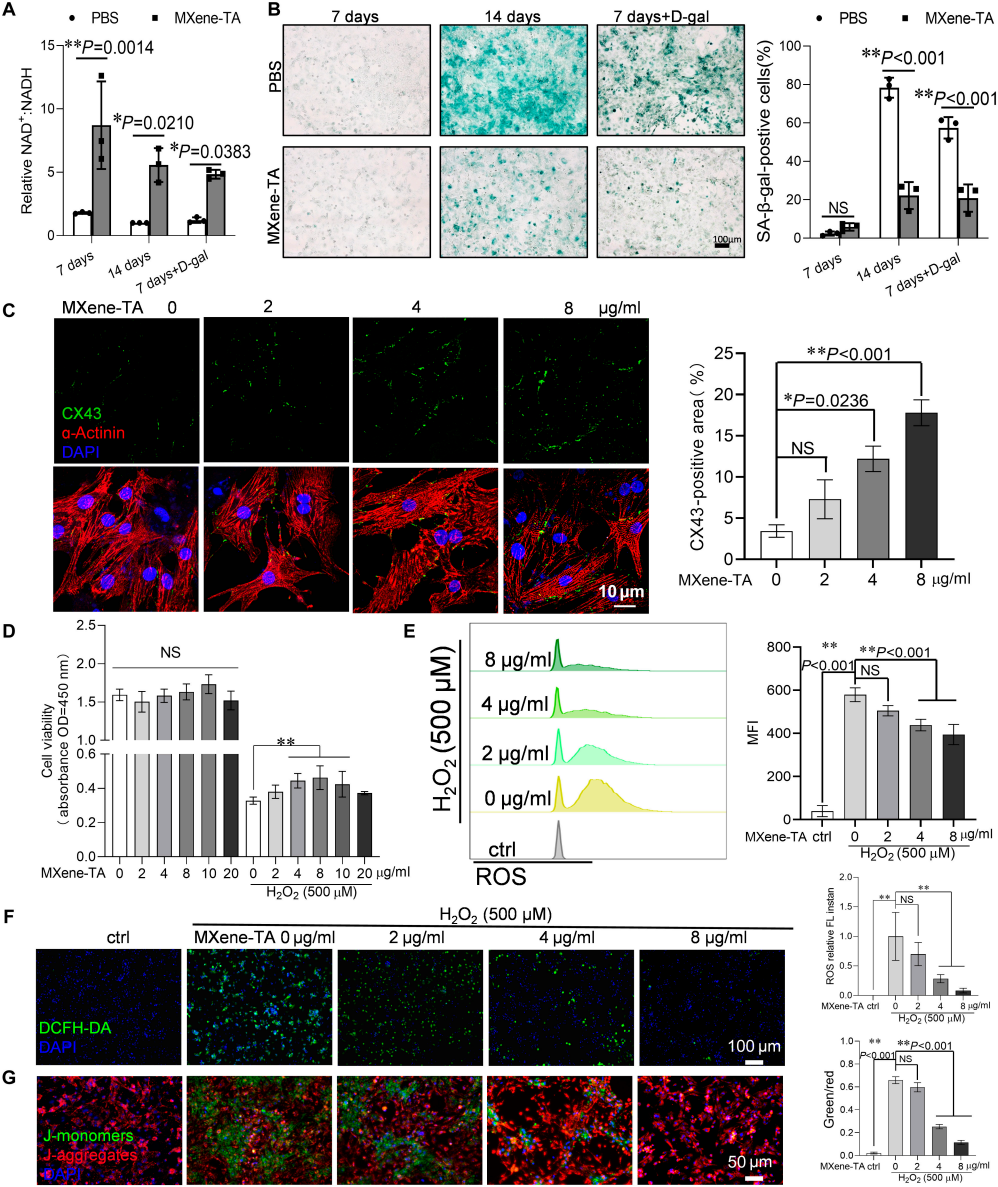
MXene-TA delays cellular senescence and reduces oxidative damage in cardiomyocytes. Primary cardiomyocytes were extracted for 7 d with d-galactose or cultured for 14 d to induce senescence with or without 8 μg/ml MXene-TA treatment for 24 h. (A) Colorimetric assay of NAD^+^/NADH ratio in cardiomyocytes (*n* = 3). (B) SA-β-gal staining for detecting cardiomyocyte senescence, representative images of cells, and quantifying SA-β-gal-positive cells. Scale bar, 100 μm; *n* = 3. (C) Immunofluorescence staining representative graphs of actinin and CX43 and quantitative analysis graph of CX43. Scale bar, 10 μm; *n* = 3. The cardiomyocytes were transduced with or without H_2_O_2_ for 6 h and then incubated with different concentrations of MXene-TA (0 to 20 μg/ml) for 24 h. Supernatants were collected, and cell viability was detected by CCK-8 (*n* = 6). (D) Total intracellular ROS in DCFH-DA staining was detected by flow cytometry (E) and fluorescence microscope (F). Scale bar, 100 μm; *n* = 3. Intracellular mitochondrial membrane potential (MMP) was observed by fluorescence microscopy. Scale bar, 50 μm; *n* = 3. (G) Quantitative data were expressed as the mean ± SD of at least 3 independent experiments. *P < 0.05; **P < 0.01.

**Fig. 4.**
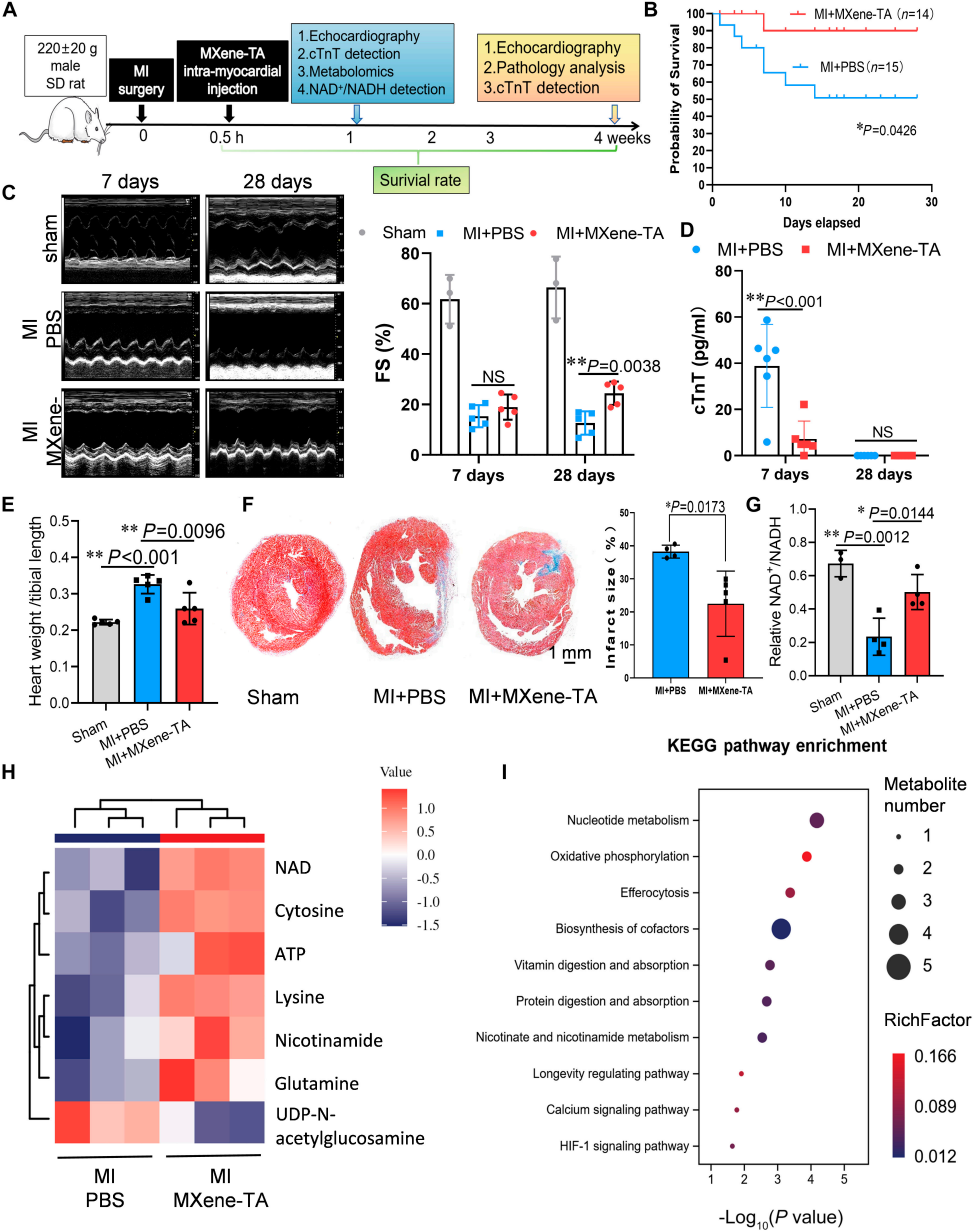
MXene-TA improves cardiac function post-MI by reprogramming energy metabolism in infarcted myocardium. (A) Schematic illustration of the experimental protocol: LAD ligation was performed to induce MI in rats, followed by a single injection of MXene-TA or PBS into the infarct border zone. Metabolic profiling was performed on day 7, and cardiac function was evaluated on day 28. (B) Kaplan–Meier survival curves showing the survival rate of MI rats in the PBS and MXene-TA groups. (C) Echocardiographic analysis of fractional shortening (FS%) at baseline, day 7, and day 28 post-MI (*n* = 3 to 5 rats per group). (D) Serum cTnT levels measured by ELISA at days 7 and 28 post-infarction (*n* = 6 rats per group). (E) Heart weight/tibia length (HW/TL) and lung weight/tibia length (LW/TL) ratios at day 28 post-MI (*n* = 5 rats per group). (F) Representative images of Masson’s trichrome staining showing infarct size at day 28 (blue, fibrosis; red, myocardium). Scale bar, 1 mm; *n* = 5 rats per group. (G) NAD^+^/NADH ratio in the infarcted region 7 d after treatment (*n* = 3 rats per group). (H) Heat map of targeted metabolomics showing differential metabolites between MXene-TA and PBS groups (*n* = 3 rats per group). (I) KEGG pathway enrichment analysis of differential metabolites. Key pathways up-regulated in the MXene-TA group include oxidative phosphorylation, nicotinate and nicotinamide metabolism, longevity-regulating pathway, calcium signaling pathway, and HIF-1 signaling. Quantitative data were expressed as the mean ± SD. *P < 0.05; **P < 0.01.

Previous experimental evidence has shown that NADH-oxidizing nanozymes effectively modulate metabolism, especially in organs with high NADH and low NAD^+^ levels. Since MI is linked to disrupted redox balance—characterized by increased NADH and decreased NAD^+^ in the damaged heart tissue—we also examined whether MXene-TA, a nanozyme with NOX-like activity, could help restore balance in MI. Within 1 to 3 d of an MI, overproduction of reactive oxygen species (ROS) causes mitochondrial oxidative stress in cardiomyocytes, leading to cellular apoptosis and necrosis [37]. NAD^+^, an essential coenzyme, plays a critical role in neutralizing free radicals and mitigating mitochondrial damage caused by oxidative stress. In this study, we investigated the regulatory effects of MXene-TA on oxidative damage in cardiomyocytes. Our findings demonstrated that treatment with MXene-TA for 24 h significantly increased the intracellular NAD^+^/NADH ratio (Fig. 3A) and reduced the number of β-galactosidase (SA-β-gal)-positive cells (Fig. 3B) in both naturally senescent (14 d) and drug-induced senescent primary cardiomyocytes. These findings indicate that MXene-TA effectively ameliorated cellular senescence, likely by restoring redox balance through NADH oxidation. To further assess the functional consequences of MXene-TA treatment, we examined the expression of α-actinin and connexin 43 (CX43), key proteins involved in cardiomyocyte cytoskeletal structure and intercellular electrical coupling, respectively [38]. As shown in Fig. 3C, MXene-TA treatment resulted in a dose-dependent up-regulation of CX43, whereas α-actinin levels remained relatively unchanged. This suggests that MXene-TA may help preserve cardiomyocyte connectivity and functionality under stress conditions. Furthermore, we evaluated the protective effects of MXene-TA on mitochondria following oxidative damage induced by hydrogen peroxide. After 8 μg/ml MXene-TA was treated on cardiomyocytes with oxidative injury, cell viability detection showed that MXene-TA significantly increased cell viability (Fig. 3D), and flow cytometry analysis showed that MXene-TA significantly decreased intracellular ROS content (Fig. 4E and F). Mitochondrial membrane potential staining showed a significant increase in membrane potential at the same concentration (Fig. 4G). These findings collectively showed that MXene-TA effectively lowered ROS levels in cardiomyocytes and maintained mitochondrial homeostasis, thus preserving cell viability. These results provided compelling evidence that MXene-TA offered a promising therapeutic approach for mitigating oxidative damage in cardiomyocytes, potentially contributing to improved outcomes in MI treatment.

### MXene-TA reprograms energy metabolism in infarcted myocardium to promote cardiac repair

Following the in vitro demonstration that MXene-TA improves redox homeostasis, mitochondrial function, and cardiomyocyte viability under oxidative stress, We subsequently evaluated the therapeutic efficacy of MXene-TA by employing a rat MI model for in vivo assessment. MI was elicited via permanent ligation of the left anterior descending (LAD) coronary artery, followed by a single injection of either MXene-TA (8 μg/ml) or PBS into the infarction border region. Cardiometabolic was assessed on day 7 post-injection, and cardiac function and remodeling were evaluated on day 28 (Fig. 4A). Survival analysis revealed that approximately 40% of rats in the PBS group died within 2 weeks post-MI, whereas the MXene-TA group exhibited significantly improved survival, with only ~10% mortality (Fig. 4B). Echocardiographic analysis revealed no difference in fractional shortening percentage (FS%) at day 7, indicating consistent infarct sizes and modeling success across groups. By day 28, FS% declined markedly in the PBS group, while MXene-TA treatment preserved systolic function (Fig. 4C). The level of serum cardiac troponin T (cTnT) elevated markedly on day 7 in PBS-treated rats. Still, they returned to baseline by day 28 in both groups (Fig. 4D), suggesting reduced acute myocardial injury in the MXene-TA group.

Given that surviving cardiomyocytes often undergo compensatory eccentric hypertrophy post-MI, contributing to adverse remodeling, we analyzed gross cardiac phenotypes. MXene-TA-treated rats exhibited reduced heart weight-to-tibia length (HW/TL) and lung weight-to-tibia length (LW/TL) ratios compared to controls (Fig. 4E and Fig. S3A), suggesting attenuation of cardiac hypertrophy and pulmonary congestion. Histological analysis confirmed that MXene-TA treatment significantly reduced infarct size without detectable pathological damage to other major organs (Fig. 4F and Fig. S3D), supporting its safety and efficacy.

To determine whether metabolic modulation underlies the cardioprotective effects of MXene-TA, we examined redox status and performed targeted metabolomics analysis in the infarcted myocardium at day 7. MXene-TA significantly increased the NAD^+^/NADH ratio compared to PBS controls (Fig. 4G), consistent with its NOX-like activity. Metabolomic profiling revealed a clear separation between groups, with significantly elevated levels of NAD^+^, cytosine, ATP, lysine, nicotinamide, and glutamine in the MXene-TA group, while uridine diphosphate (UDP)-N-acetylglucosamine levels were decreased (Fig. 4H and Fig. S3B). Kyoto Encyclopedia of Genes and Genomes (KEGG) pathway enrichment analysis indicated up-regulation of oxidative phosphorylation, nicotinate and nicotinamide metabolism, the longevity-regulating pathway, calcium signaling, and hypoxia-inducible factor-1 (HIF-1) signaling (Fig. 4I and Fig. S3C). Collectively, these results demonstrate that a single administration of MXene-TA into the infarcted myocardium during LAD ligation reprograms local energy metabolism, enhances redox balance, and promotes myocardial repair, leading to improved survival and cardiac function post-MI.

### MXene-TA modulates the senescence of ADSCs by regulating the NAD^+^/NADH ratio

**Fig. 5.**
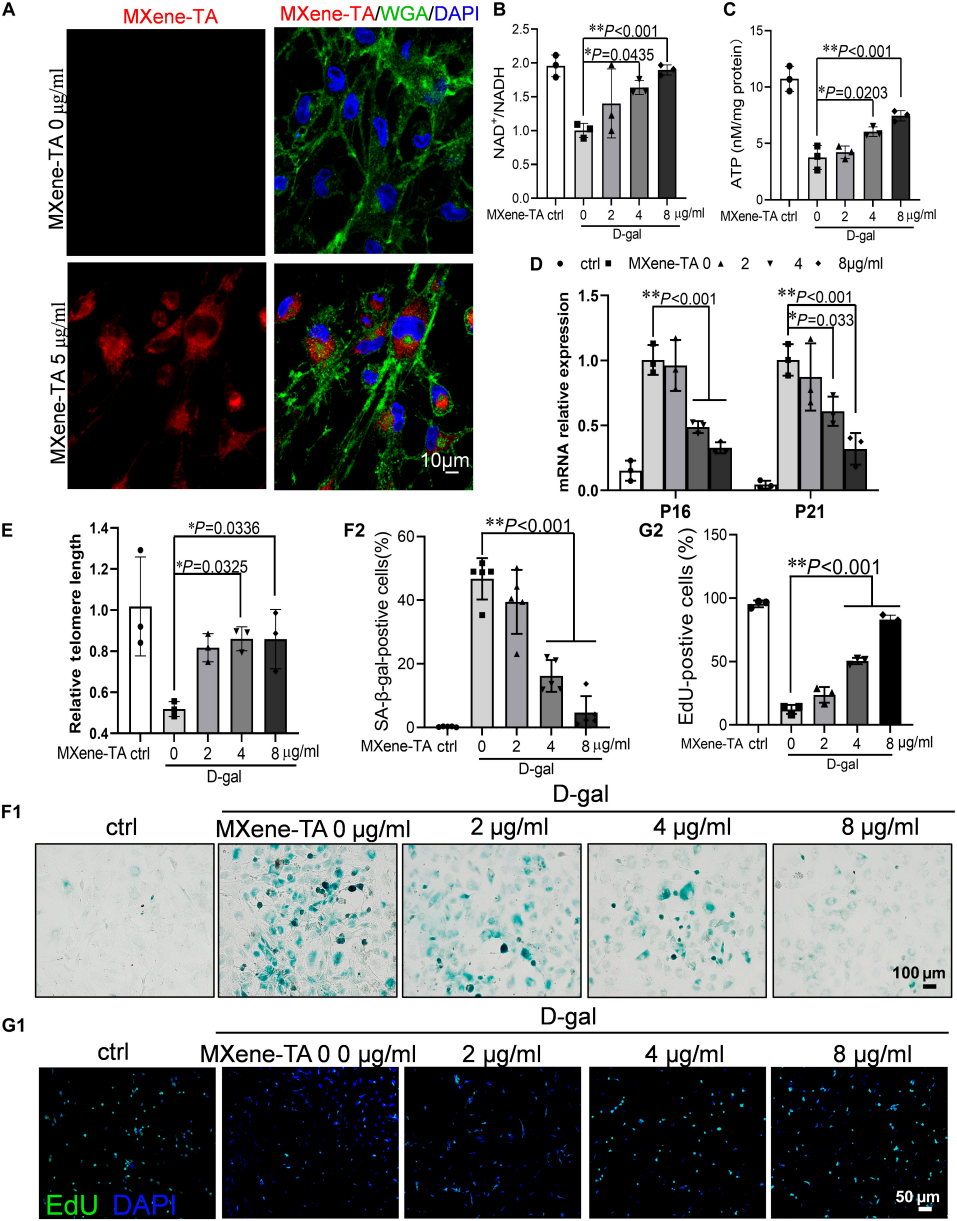
MXene-TA regulates NAD^+^/NADH ratio and delays mesenchymal stem cell senescence. (A) Fluorescent confocal laser scanning microscopic images of ADSCs after incubation with MXene-TA within 6 h; red fluorescent images of rhodamine-labeled MXene-TA; green fluorescent images of ADSCs stained by WGA; blue fluorescent images of the nucleus. Scale bar, 10 μm. (B to G) ADSC cells were treated with different concentrations of MXene-TA (0 to 8 μg/ml) for 24 h with or without d-galactose. (B) Enzymatic activity of MXene-TA in ADSC cells, *n* = 3. (D) Relative quantitative PCR for telomere length (*n* = 3). (E) RT-qPCR detection of senescence-related genes P16 and P21 mRNA expression (*n* = 3). (F1 and F2) Representative images of SA-β-gal-stained cells and quantification of SA-β-gal-positive cells. Scale bar, 50 μm; *n* = 5. (G1 and G2). EdU staining of ADSCs. Scale bar, 50 μm; *n* = 3. All data are presented as mean ± SD of at least 3 independent experiments. *P < 0.05, **P < 0.01.

Stem cell grafting is considered a prospective approach to treat MI. However, low cell survival and reduced functionality within the infarcted microenvironment continue to be major challenges. Building on our previous findings that MXene-TA supports redox homeostasis, we hypothesized that MXene-TA could improve the metabolic microenvironment of ADSCs and increase their viability in ischemic tissue. To explore whether MXene-TA can catalyze the conversion of NADH to NAD^+^ inside ADSCs, we first assessed its cellular uptake efficiency. MXene-TA was labeled with rhodamine for visualization, while the ADSC membrane was counterstained with wheat germ agglutinin (WGA) to localize the cell boundary. Confocal microscopy analysis confirmed successful internalization of MXene-TA, with predominant accumulation in the cytoplasmic compartment (Fig. 5A). To determine whether MXene-TA can enter the cell through endosomal transport, we labeled the lysosomes and MXene-TA with fluorescence. We then observed their localization within the cell over a period of 6 h. The results showed that after 2 h, most of MXene-TA entered the lysosomes, and after 6 h, most of it escaped from the lysosomes, demonstrating the phenomenon of lysosome escape (Fig. S4A).

To determine its redox catalytic function, we next quantified the intracellular NAD^+^/NADH ratio following MXene-TA treatment. As shown in Fig. 5B, MXene-TA significantly elevated the NAD^+^/NADH ratio in a dose-dependent manner in d-galactose–induced senescent ADSCs, indicating enhanced NADH oxidation. Given the established relationship between NAD^+^/NADH balance and cellular senescence, we further evaluated the anti-aging effects of MXene-TA in ADSCs. Our results showed that MXene-TA treatment alleviated senescence through multiple mechanisms. It markedly increased intracellular ATP production (Fig. 5C), indicative of improved mitochondrial energy metabolism, and significantly delayed telomere shortening—a hallmark of replicative senescence (Fig. 5D). Moreover, the expression of key senescence-associated markers p16 and p21 was significantly reduced upon MXene-TA treatment (Fig. 5E). Consistently, the percentage of SA-β-gal–positive cells was notably decreased (Fig. 5F1 and F2), indicating reduced cellular senescence. Additionally, 5-ethynyl-2′-deoxyuridine (EdU) incorporation assays revealed that MXene-TA significantly enhanced ADSC proliferation compared with control groups (Fig. 5G). Together, these findings demonstrate that MXene-TA not only enhances intracellular NAD^+^/NADH redox balance but also improves mitochondrial bioenergetics, delays senescence progression, and promotes the proliferative capacity of ADSCs, suggesting its therapeutic potential in enhancing stem cell viability and function for myocardial recovery.

### Preparation and characterization of MXene-TA- and ADSC-loaded conductive hydrogels

**Fig. 6.**
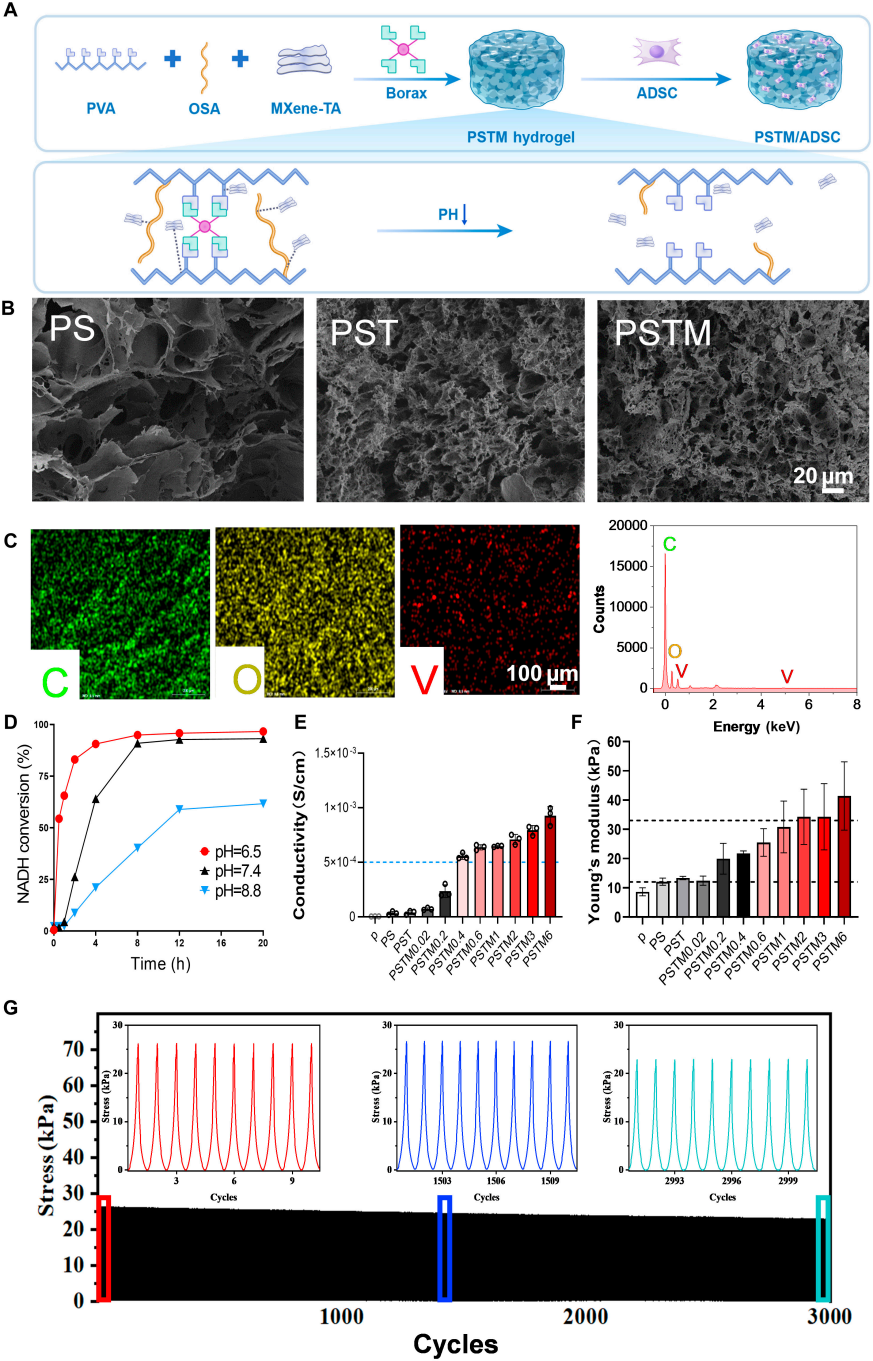
Characterization of different scaffolds. (A) Fabrication of the NAD^+^/NADH metabolic reprogramming hydrogel. (B) SEM images of PS, PST, and PSTM scaffolds. (C) SEM-EDS elemental mappings (left: C element; middle: O element; right: V element) and EDS spectral analysis (right) of PSTM scaffold. (D) The NADH clearance of PSTM hydrogel was measured by colorimetry in different pH solutions. (E) Conductivities of different scaffolds (*n* = 3). Young’s modulus (F) of PS, PST, and PSTM(x) scaffold (*n* = 3). (G) Cyclic compression tests of PSTM0.6 scaffolds. Each sample was compressed to a strain of 50% at a 150 mm/min rate for 3,000 cycles.

To achieve both metabolic reprogramming and electrical coupling in the infarcted myocardium, we engineered a multifunctional, responsive hydrogel—called PSTM/ADSC—that includes PVA, OSA, borax, tannic acid, MXene-TA, and ADSCs. As shown in Fig. 6A, OSA was created by oxidizing sodium alginate with sodium periodate and acts as the backbone polymer. Borax was added to form borate ester bonds, giving the matrix pH sensitivity. Tannic acid improved the hydrogel’s adhesiveness and cross-linking density, while MXene-TA provided both catalytic bioactivity and electrical conductivity. Finally, ADSCs were encapsulated within the PSTM hydrogel to form a composite system capable of regulating NAD^+^/NADH levels and enabling electrical integration.

SEM revealed that all hydrogel variants (PS, PST, and PSTM) possessed typical porous microstructures (Fig. 6B). The average pore diameter was reduced from 30 to 60 μm in PS to ~10 μm in PST and PSTM hydrogels after tannic acid incorporation—dimensions suitable for stem cell attachment and proliferation [39]. Energy-dispersive x-ray spectroscopy (EDS) further confirmed the uniform distribution of vanadium—a characteristic element of MXene-TA—within the PSTM scaffold, verifying successful integration (Fig. 6C). The pH-responsive behavior of PSTM was assessed by immersing hydrogels in NADH solutions at pH 6.5, 7.4, and 8.8. As shown in Fig. 6D, NADH oxidation activity was significantly enhanced under acidic conditions, suggesting efficient pH-triggered release of MXene-TA in the ischemic microenvironment.

Given that conductive hydrogels can facilitate electrical signal propagation in infarcted myocardium [40,41], we next evaluated the electrochemical properties of PSTM using cyclic voltammetry (CV). The conductivity of PST hydrogel (without MXene-TA) was 3.924 × 10^−5^ S/cm, whereas incorporation of 4 mg/ml MXene-TA (PSTM0.4%) elevated the conductivity to >5.0 × 10^−4^ S/cm, within the physiological conductivity range of native cardiac tissue (5.0 × 10^−4^ to 1.6 × 10^−2^ S/cm) (Fig. 6E). Myocardial tissue engineering scaffolds with appropriate mechanical performance can supply critical structural supports for repairing hard and brittle hearts following MI [42]. As shown in Fig. S5A and B, under vertical pressure, no significant deformation was observed in appearance. The elastic modulus of PST was measured at 12 kPa, increasing progressively with MXene-TA concentration, reaching >40 kPa at 6% MXene-TA (Fig. 6F), which is well within the physiological range of myocardial stiffness (5 to 500 kPa) [43]. Furthermore, all scaffold variants maintained mechanical stability under cyclic strain, showing minimal stress loss after 3,000 cycles at 50% compression (Fig. 6G). Considering that ADSCs optimally maintain their stemness on substrates with stiffness ranging from 11 to 34 kPa [44], we selected 4 MXene-TA concentrations (2, 4, 6, and 10 mg/ml; i.e., PSTM0.2%, 0.4%, 0.6%, and 1%) for downstream in vitro studies. Next, we performed rheological experiments to evaluate the stability of the hydrogels. The internal structure of the hydrogels was characterized by monitoring the storage modulus (*G*′) and loss modulus (*G*″). We further examined the rheological behavior of PS, PST, and PSTM hydrogels. Across the entire angular frequency range (ω = 0.01 to 10 rad/s), the *G*′ values consistently exceeded the *G*″ values, indicating that the hydrogels maintained an intact and stable network structure. In addition, the gelation time, stability, and self-healing ability of the PSTM hydrogel were investigated. As shown in Fig. S5C to F, the PSTM hydrogel underwent rapid gelation, exhibited structural stability after formation, and demonstrated pronounced self-healing properties. In summary, the constructed hydrogel exhibited excellent microenvironmental responsiveness to MI and possesses mechano-electrical morphological characteristics that supported stem cell self-proliferation.

Given that conductive hydrogels can enhance electrical coupling between cardiomyocytes and potentially repair electrical disturbances in MI [45], we further tested whether the conductive hydrogel environment could enhance electrical synchronization between CMs. PSTM hydrogels with up to 6 mg/ml MXene-TA exhibited excellent biocompatibility (Fig. S6A). Analysis of Ca^2+^ transients, a proxy for action potential propagation, revealed strong and synchronized calcium fluxes in CMs cultured on PSTM0.6% hydrogels, while nonconductive PST hydrogels exhibited weak and asynchronous signals (Fig. S6B and Movie S1). These findings suggest that MXene-TA significantly enhances intercellular electrical coupling and cardiomyocyte (CM) synchronization. To evaluate the anti-senescence capacity of MXene-TA in a 3-dimensional (3D) hydrogel context, we conducted SA-β-gal staining of ADSCs cultured within PSTM hydrogels. Notably, PSTM0.6% significantly reduced cellular senescence (Fig. S6C), consistent with previous 2D culture data. These findings collectively demonstrate that MXene-TA-loaded PSTM hydrogels possess tunable electrical and mechanical properties, exhibit pH-triggered catalytic release, and promote ADSC viability and anti-senescence effect, thereby offering a multifunctional platform suitable for cardiac tissue engineering.

### PSTM hydrogel via CX43 to increase the expression and secretion of SDF-1 protein

**Fig. 7.**
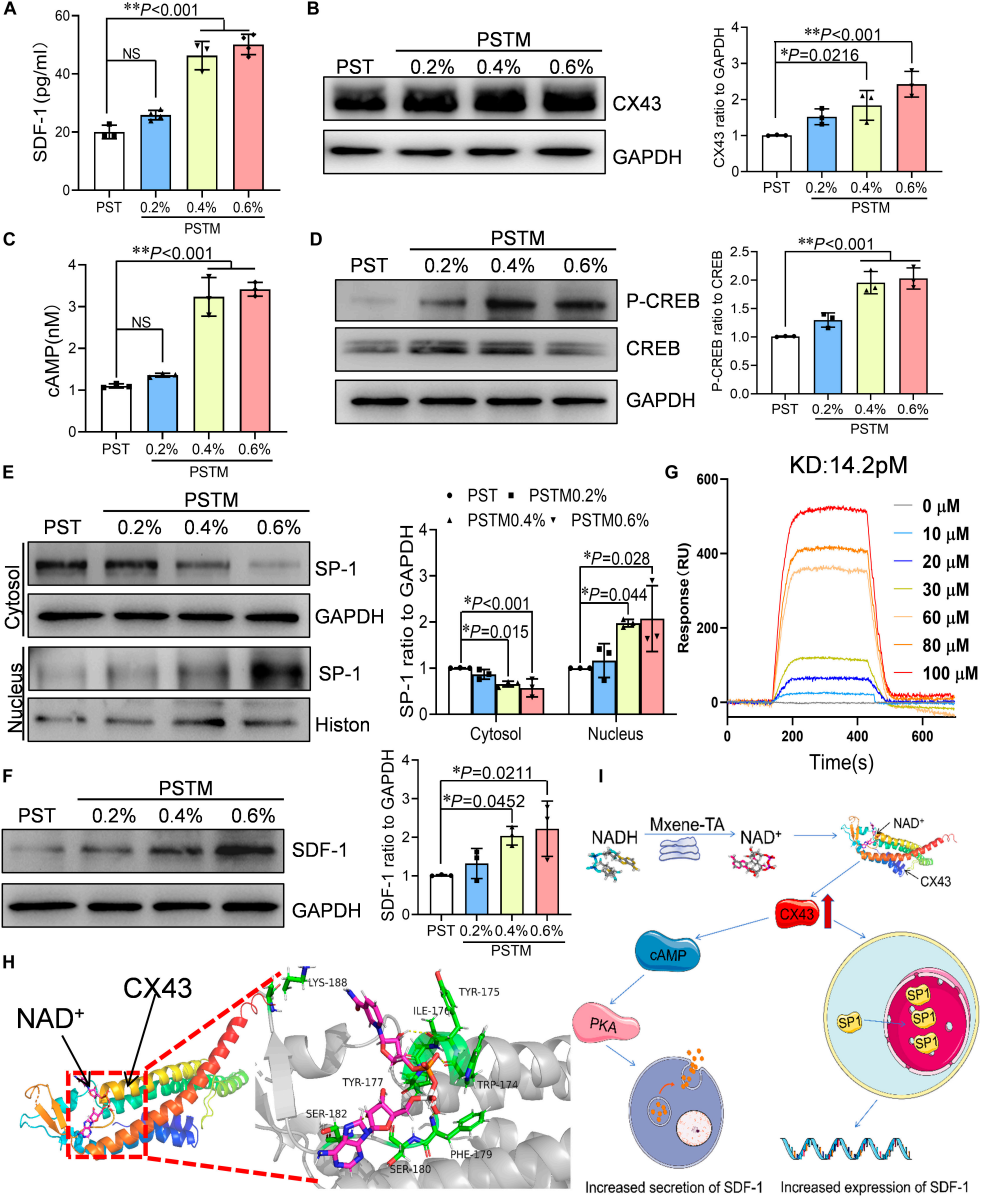
PSTM hydrogels increase SDF-1 protein expression and secretion via CX43. ADSCs were cultured on different hydrogels for 3 d; the supernatants were collected, and the ELISA assay was used to detect the expression of SDF-1 (A) and cAMP (C) (*n* = 3). Total proteins were extracted after the cells were collected, and Western blotting was used to detect intracellular expression of CX43 (B), CREB, phosphorylated CREB (D), and SDF-1 (F) proteins (*n* = 3). Nuclear and plasma proteins were extracted after cell collection, and Western blotting detected the expression of SP-1 in the cytoplasm and nucleus (*n* = 3) (E). (G) Surface plasmon resonance (SPR) was used to detect the binding ability of NAD^+^ to CX43 and calculate the dissociation constant (*K*_D_). (H) Molecular dynamics predicted the binding mode and binding site of NAD^+^ to CX43. (I) Schematic diagram of the mechanism. Quantitative data were expressed as the mean ± SD of at least 3 independent experiments. *P < 0.05; **P < 0.01.

Subsequently, we aimed to clarify the specific mechanism by which MXene-TA alters the fate of ADSCs by influencing NAD^+^ levels. Cytokines are crucial components of the ADSC microenvironment, influencing ADSC characteristics and differentiation potential through cell-to-cell interactions and the secretion of signaling molecules [46]. To explore this, ADSCs were cultured on PSTM hydrogel, and the secretion of 13 distinct cytokines was evaluated. As depicted in Fig. 7A and Fig. S7A, stromal cell-derived factor-1 (SDF-1) secretion exhibited a concentration-dependent increase with rising MXene-TA levels, whereas the secretion of other cytokines, including stem cell factor-1 (SCF-1), insulin-like growth factor-1 (IGF-1), transforming growth factor-β (TGF-β), epidermal growth factor (EGF), vascular endothelial growth factor (VEGF), leukemia inhibitory factor (LIF), macrophage colony-stimulating factor (M-CSF), interleukin-6 (IL-6), Ang-1, hepatocyte growth factor (HGF), fibroblast growth factor (FGF), and platelet-derived growth factor-AA (PDGF-AA), remained unchanged.

Studies have shown that secretion of SDF-1 by human bone marrow-derived adipose stem cells (BADSCs) depends on cell–cell contact, which is mobilized by interstitial gap junctions CX43 [47,48]. This process involved calcium conductance activating the protein kinase A (PKA) signaling pathway, which induced guanosine triphosphatase (GTPase) RalA-mediated SDF-1 secretion. In addition, SDF-1 transcription is regulated by CX43 through the nuclear localization of the transcription factor Sp1 [49]. Based on these findings, we hypothesized that MXene-TA enhances NAD^+^ levels within ADSC, thereby up-regulating CX43 to promote SDF-1 expression and secretion. To test this hypothesis, we initially employed Western blotting to assess the effect of CX43 protein expression in ADSCs treated with MXene-TA. The results revealed a significant concentration-dependent increase in CX43 protein levels (Fig. 7B). Similarly, MXene-TA augmented CX43 protein expression in cardiomyocytes (Fig. 3C).

Further investigations were conducted to determine whether MXene-TA enhanced SDF-1 secretion via the adenosine 3′,5′-monophosphate (cAMP)/PKA pathway. Figure 7C and D illustrates that MXene-TA significantly elevated cAMP levels in ADSC and enhanced PKA activity, leading to the phosphorylation and activation of cAMP response element–binding protein (CREB), thereby increasing SDF-1 secretion. Concurrently, Western blot analysis demonstrated that MXene-TA facilitated the translocation of the transcription factor Sp1 from the cytoplasm to the nucleus in ADSC (Fig. 7E), resulting in a marked up-regulation of SDF-1 protein expression (Fig. 7F). To further elucidate how NAD^+^ regulates CX43 to augment its expression, we considered that NAD^+^, as a small molecule, typically binds to proteins to modulate their structure, thereby enhancing stability and preventing degradation. Surface plasmon resonance (SPR) analysis confirmed that NAD^+^ stably binds to CX43 with a dissociation constant (*K*_D_) of 14.2 pM (Fig. 7G). Molecular dynamics and molecular docking simulation identified specific binding sites on CX43, including Lys^188^, Tyr^175^, Ile^176^, Tyr^177^, Trp^174^, Phe^179^, Leu^181^, Gly^178^, Ser^182^, and Ser^180^ (Fig. 7H and Fig. S8A). The binding interactions were characterized by van der Waals forces, hydrogen bonding, carbon–hydrogen bonding, and pi–pi–T-shaped and pi–cation interactions (Fig. S8B and C). Molecular dynamics analysis showed the number of hydrogen bonds formed between CX43 protein and NAD+, and the root mean square deviation (RMSD) of the CX43–NAD+ complex indicated that the CX43 protein remained stable when bound to NAD+ (Fig. S8D to F).

In summary, our findings suggested that MXene-TA functions as a NOX in ADSC, generating NAD^+^. The binding of NAD^+^ to CX43 enhanced CX43 protein levels, promoting SDF-1 secretion by activating the cAMP/PKA pathway and facilitating Sp1 nuclear translocation to up-regulate SDF-1 expression (Fig. 7I). This dual mechanism underscored the role of MXene-TA in regulating ADSC fate.

### PSTM/ADSC hydrogel promoted mesenchymal stem cell survival and MI repair

**Fig. 8.**
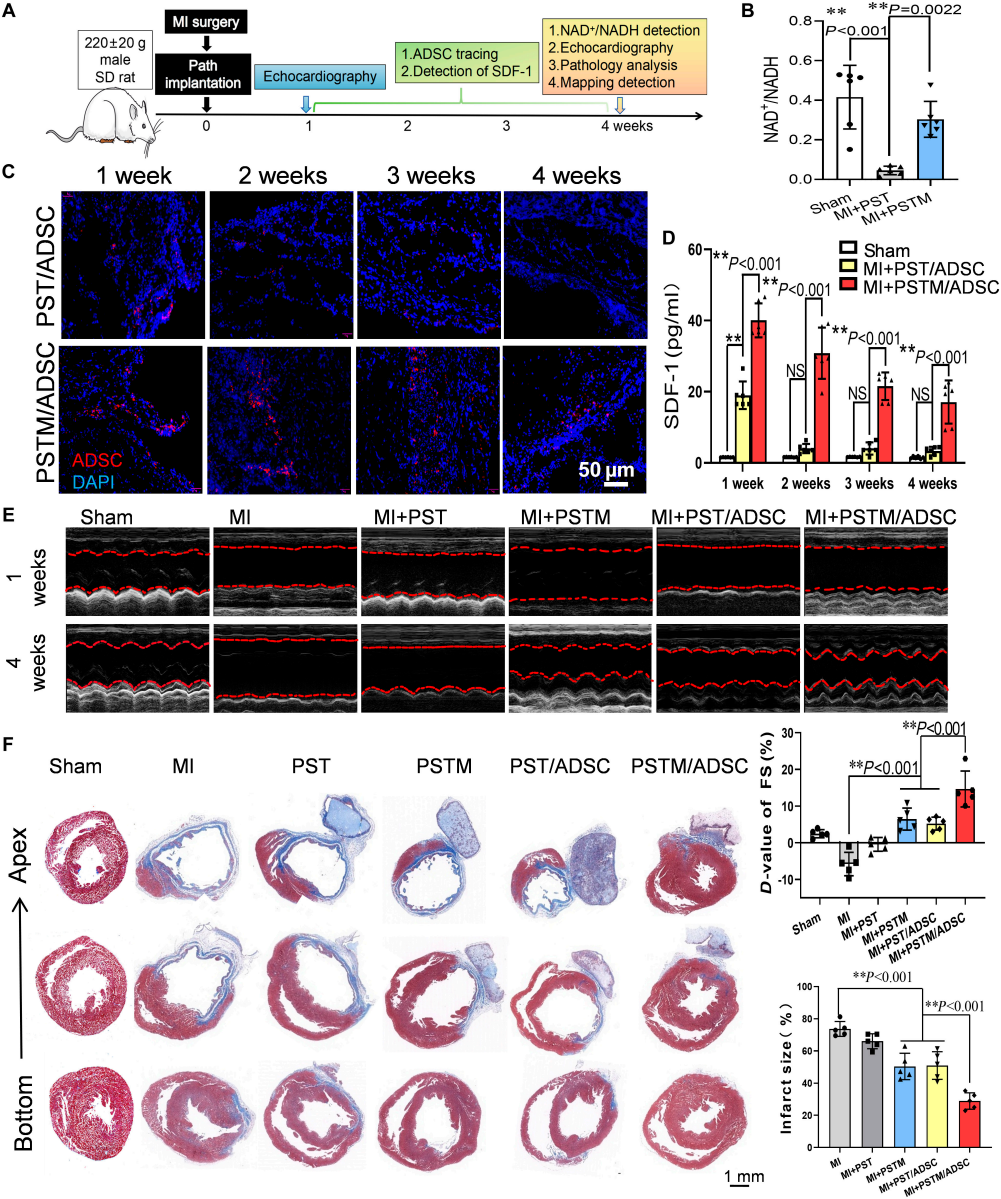
Mesenchymal stem cell survival and MI repair by PSTM/ADSC hydrogels. (A) Experimental design. (B) Myocardial infarcted rats were implanted with different hydrogel scaffolds, and the NAD^+^/NADH ratio of the infarcted tissues of rats in each group was determined by colorimetric method after 4 weeks (*n* = 6 rats per group). (C) After staining ADSCs on PST and PSTM hydrogels using live cell tracking dye, the hydrogels were implanted into infarcted rats. The hearts were harvested and sectioned within 1 to 4 weeks, and ADSC cell survival was observed by fluorescence microscopy. (D) Myocardial infarcted rats were implanted with different scaffolds, and ELISA measured the serum SDF-1 levels in each group of rats at the indicated time points (*n* = 6 rats per group). (E) Echocardiographic images of 1-week (above) and 4-week (bottom) post-MI injury in the different groups. Representative parameters of left ventricular function based on echocardiography of each group at 1 and 4 weeks after MI injury (*n* = 6 rats per group). (F) Mason’s staining displayed the fibrous tissue (blue) and myocardium (red) of sections from the bottom to the apex of hearts 28 d post-MI in different groups. Scale bars, 1 mm. Right panel, statistical analysis of infarct size of the infarcted heart in different groups (*n* = 5 rats per group). All data are presented as mean ± SD. *P < 0.05; **P < 0.01.

After identifying the critical role of MXene-TA in delayed ADSC senescence and attenuated oxidative stress in CMs, we next investigated whether the PSTM/ADSC metabolic reprogramming hydrogel can act as a possible treatment strategy for MI. In vitro experiments showed that PSTM0.6% exerted superior biological effects, so in vivo experiments were performed using equal concentrations of MXene-TA added to PST hydrogel. A rat MI model was established to evaluate this, and various hydrogel formulations were implanted to assess ADSC survival, SDF-1 secretion, and cardiac functional recovery over 4 weeks (Fig. 8A). Notably, the NAD^+^/NADH ratio in myocardial tissue remained significantly elevated 4 weeks post-implantation of PSTM hydrogel (Fig. 8B), suggesting sustained metabolic reprogramming. Fluorescence microscopy of ADSCs labeled with a live-cell dye and implanted into the infarcted region revealed that ADSCs survived for only 1 week in the PST hydrogel group, while their survival extended to at least 4 weeks in the PSTM hydrogel group (Fig. 8C). Enzyme-linked immunosorbent assay (ELISA) analysis of serum SDF-1 levels demonstrated that the PSTM/ADSC hydrogel group exhibited significantly higher SDF-1 secretion compared to the PST/ADSC hydrogel group (Fig. 8D). This finding indicated that PSTM hydrogel provides a favorable microenvironment for ADSC survival.

Echocardiographic assessments further confirmed that implantation of PSTM, PST/ADSC, and PSTM/ADSC hydrogels notably improved cardiac function post-MI, with the PSTM/ADSC hydrogel group outperforming the other groups (Fig. 8E and Fig. S9A). Histological analysis using Masson’s staining showed that both PSTM and PST/ADSC hydrogels decreased the size of fibrotic areas compared to the MI group, with the PSTM/ADSC hydrogel showing the most pronounced effect (Fig. 8F). Additionally, H&E staining indicated that the PSTM/ADSC hydrogel group exhibited the least inflammatory cell infiltration, further supporting its anti-inflammatory and reparative properties (Fig. S9B). Collectively, these results suggested that the PSTM/ADSC metabolic reprogramming hydrogel functions as a NOX in myocardial tissue, promoting long-term ADSC survival and enhancing cardiac repair following MI.

### PSTM/ADSC hydrogels regulated cardiac electrical conduction, inflammation, and neovascularization after MI

**Fig. 9.**
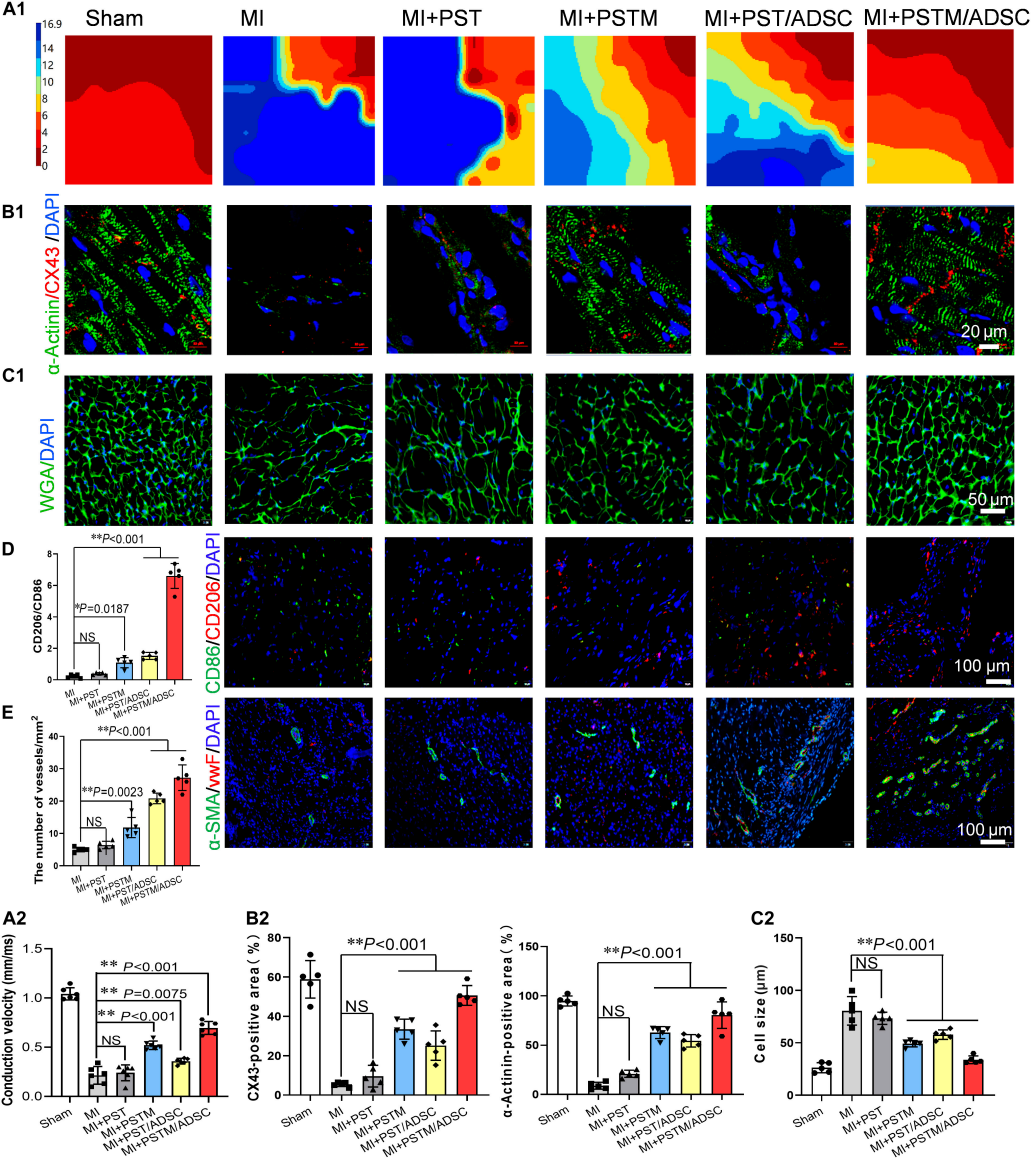
Repair effects of PSTM/ADSC patch in rat MI models after implantation for 28 d. (A1) Representative epicardial activation maps were recorded by a 64-channel electromapping system from the healthy myocardium to the infarcted myocardium in different groups. Ruler: red represented the first activation, blue represented the last activation, and numbers indicated activation time (ms). (B1) Immunofluorescence staining of cardiac-specific proteins (α-actinin: green, CX43: red) in different groups’ infarcted area of hearts 28 d post-MI. (C) Immunofluorescence staining of WGA (green) to display the boundaries of CMs within the border area. (D and E) Immunofluorescence staining of CD86 (green)/CD206 (red) (D) and α-SMA (green)/vWF (red) (E) within the infarcted area in different groups, statistical analysis of microvessel densities (*n* = 5 rats per group), and the ratio of M2/M1 macrophages (*n* = 5 rats per group) within the infarcted region in different groups. (A2) Conduction velocities in different groups were calculated using epicardial activation maps (*n* = 5 rats per group). (B2) Quantitative analysis of α-actinin- and CX43-positive area in different groups (*n* = 5 rats per group). (C2) Statistical analysis of CM size within the infarcted region in different groups based on WGA staining (*n* = 5 rats per group). All data are presented as mean ± SD. *P < 0.05; **P < 0.01.

In our previously published research, we demonstrated that conductive cardiac patches can effectively enhance electrical conduction integration in infarcted hearts [41,50]. To further evaluate the impact of different myocardial patches on electrical integration, we used an electrocardiogram system to measure the velocity of conduction and amplitude of local electrical potentials in the heart between healthy and infarcted tissue 28 d after surgery. As shown in Fig. 9A1 and A2 and Fig. S9C, electrical conduction was significantly delayed in the MI region in the MI and PST collagen gel groups. In contrast, implantation of PSTM, PST/ADSC, and PSTM/ADSC collagen gels significantly accelerated electrical conduction from normal myocardium to the MI zone. Additionally, the PSTM/ADSC group exhibited the highest conduction velocity and the largest local potential amplitude, indicating its stronger ability to promote electrical conduction in the infarct heart. CX43 protein is responsible for intercellular communication and electrical impulse transmission in the left ventricle [51]. To assess the level of electrical integration, we tested the CX43 protein expression in cardiomyocytes within the infarcted area following patch implantation. As shown in Fig. 9B, CX43 protein expression was notably reduced in the MI and PST patch groups but was abundantly present in the PSTM/ADSC group. Additionally, α-actinin-positive myocardial tissue in the infarcted region of the PSTM/ADSC group was well-organized, with densely distributed CX43 protein (Fig. 9B1 and B2).

Next, we stained the edges of CM with WGA (a glycoprotein membrane marker of CM) to observe the effect of myocardial patches on myocardial hypertrophy (Fig. 9C1). Compared with the Sham group, the size of CM in the MI and PST hydrogel groups significantly increased, indicating obvious cardiac tumescence. In contrast, CM size was significantly reduced in the PSTM, PST/ADSC, and PSTM/ADSC hydrogel groups, with the smallest CM size observed in the PSTM/ADSC group (Fig. 9C2). These findings show that the PSTM/ADSC myocardial patch effectively inhibits CM hypertrophy and attenuates ventricular remodeling. Macrophage polarization plays a critical role in post-MI repair, with M1-type macrophages exacerbating tissue damage and M2-type macrophages promoting tissue repair and angiogenesis. Immunofluorescence staining of cardiac sections labeled with CD206 (M2 marker) and CD86 (M1 marker) revealed that M1-type macrophages were widely infiltrated in the MI group, while M2-type macrophages were sparse. In contrast, the PSTM/ADSC group exhibited a significantly higher M2/M1 ratio, indicating a favorable shift toward a reparative macrophage phenotype (Fig. 9D). Effective myocardial repair post-MI also requires robust vascular reconstruction to provide adequate nutrients, rescue ischemic myocardium, and restore cardiac function [52,53]. Immunofluorescence staining of myocardial slices using labels for microvessels [von Willebrand factor (vWF)] and small arteries [vWF and α-smooth muscle actin (α-SMA)] showed that the PSTM/ADSC group had markedly more microvessels and small arteries than the other groups (Fig. 9E).

**Fig. 10.**
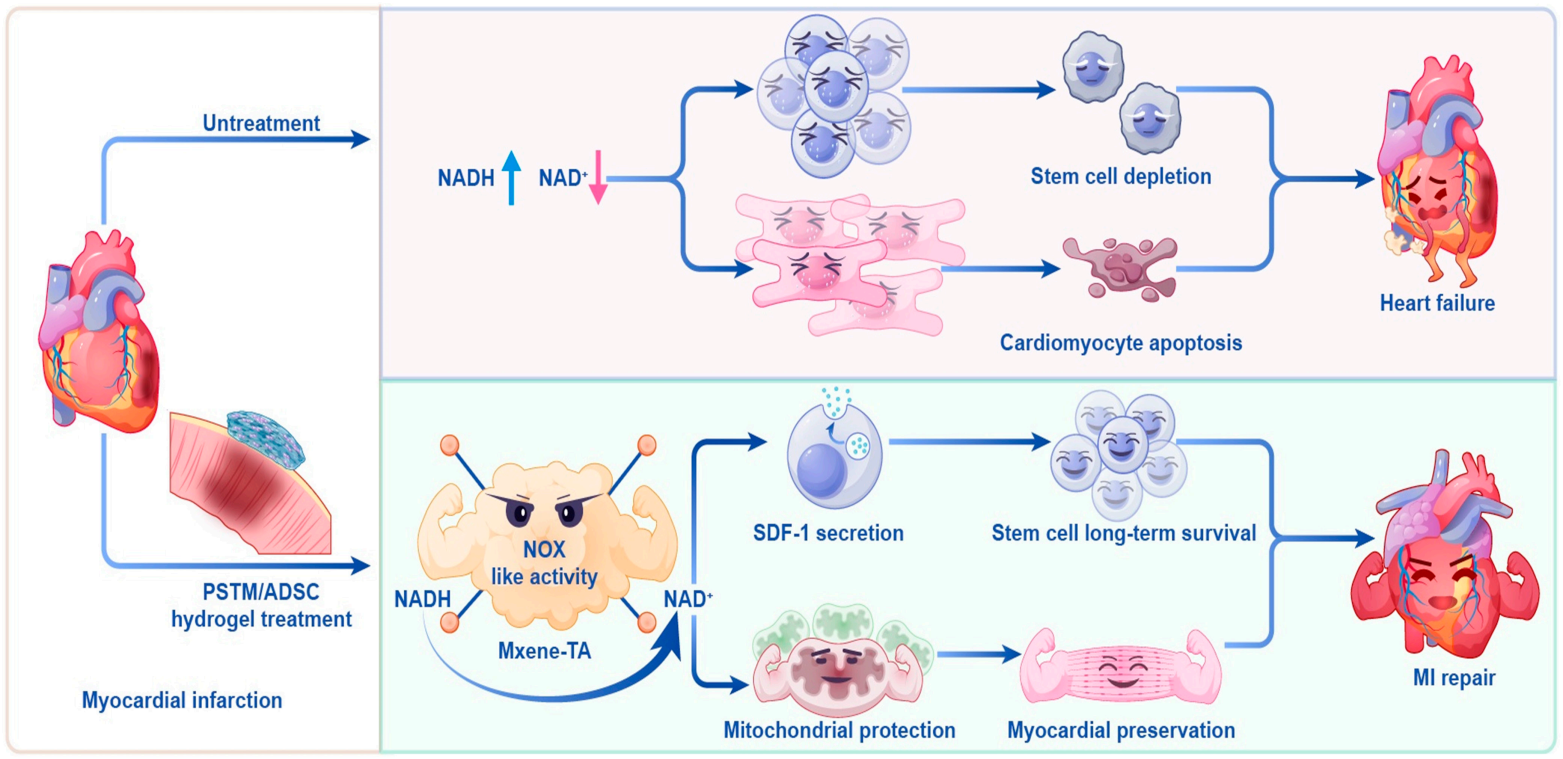
Schematic diagram of PSTM/ADSC treatment for MI: In the microenvironment of MI, increased NAD^+^ depletion is accompanied by increased NADH levels, and as the infarction progresses, intrinsic stem cells are depleted, and cardiomyocytes suffer apoptosis and necrosis. Implanting PSTM/ADSC hydrogel into infarcted rats restored NAD^+^/NADH metabolism malfunction in the infarcted area, promoted stem cell survival by increasing cell secretion of SDF-1, and, on the other hand, maintained the mitochondrial homeostasis and reduced oxidative damage of cardiomyocytes, which resulted in the effect of MI repair.

In summary, the PSTM/ADSC myocardial patch promoted electrical integration in the infarcted zone, inhibited left ventricular remodeling, modulated the inflammatory response, and enhanced vascular regeneration. Unlike traditional materials that primarily mimic the extracellular matrix (ECM) ecosystem to support MI repair [54], the PSTM/ADSC patch offers a more advanced and multifunctional approach. In addition to establishing a biomimetic cardiac ECM ecosystem, the PSTM/ADSC patch leveraged MXene-TA to reprogram the MI microenvironment through NAD^+^/NADH metabolic modulation. This metabolic reprogramming not only extended ADSC survival but also mitigated oxidative stress damage in cardiomyocytes, thereby significantly enhancing the therapeutic efficacy of MI repair (Fig. 10).

## Discussion

MI is characterized by irreversible ischemic injury and subsequent adverse ventricular remodeling, largely driven by metabolic disruption and oxidative stress. Despite the promise of ADSC-based therapies, their clinical efficacy remains limited due to poor cell survival and integration in the hostile infarct microenvironment. In this study, we developed a NOX-mimetic nanozyme (MXene-TA) and demonstrated its ability to reprogram NAD^+^/NADH metabolism, reduce cellular senescence, and enhance myocardial repair both in vitro and in vivo.

One of the key findings of our study is that MXene-TA mimics bacterial NOX activity by directly converting NADH to NAD^+^, which helps restore intracellular redox balance. Unlike traditional NAD^+^ precursor supplementation strategies that depend on inefficient metabolic pathways or face limitations due to poor bioavailability and off-target effect toxicity [24], MXene-TA provides a catalytic approach to elevate NAD^+^ levels. We confirmed that MXene-TA increased NAD^+^/NADH ratios, enhanced mitochondrial function, and suppressed senescence markers in both cardiomyocytes and ADSCs. Notably, systemic administration in aged mice revealed its multi-organ anti-aging efficacy, particularly in the heart, liver, and spleen, suggesting broad therapeutic potential beyond cardiac repair. To clarify the organ-selective anti-aging effects of MXene-TA, we integrate 4 key mechanisms: First, tissue penetration and retention differ due to MXene-TA’s physicochemical properties and organ-specific vascular/structural barriers—highly vascularized organs with leaky capillaries (heart, liver, spleen, e.g., liver sinusoids) facilitate nanoparticle extravasation, while the lung (alveolar–capillary barrier) and kidney (glomerular filtration with dense collagen IV in glomerular basement membrane) restrict access via tight junctions or filtration, reducing bioavailability [55]. Second, organ-specific NAD^+^ metabolism plays a role: The heart and liver rely on the salvage pathway (high nicotinamide phosphoribosyltransferase expression); the kidney depends on de novo synthesis (less sensitive to boosters); and the spleen, with high immune cell turnover, up-regulates NAMPT during activation, enhancing NAD^+^ sensitivity, unlike the lung (low steady-state immune turnover) [56]. Third, microenvironmental factors: moderate hypoxia (pO₂ ~10 to 50 mmHg) in the heart/liver stabilizes HIFs and induces oxidative stress, where MXene-TA’s antioxidant and NAD^+^-boosting effects synergize to mitigate senescence; the lung (alveolar pO₂ ~100 mmHg) and renal cortex (pO₂ ~40 to 60 mmHg) have higher baseline oxygen, reducing HIF stabilization and NAD^+^-dependent repair [57]. Fourth, cell-type sensitivity: cardiomyocytes (SIRT1-high) and hepatocytes (lipid metabolism-dependent NAD^+^) respond robustly to NAD^+^ elevation, while splenic immune cells (cytokine/antigen presentation linked to NAD^+^) are also sensitive; lung alveolar epithelial cells and renal tubules prioritize ion transport/TGF-β signaling over NAD^+^-mediated repair, limiting responsiveness [58].

In the context of MI, the infarct microenvironment is acidic, hypoxic, and metabolically dysregulated [59]. We found that injecting MXene-TA locally into the infarction marginal area could alter nucleotide metabolism, coenzyme factor synthesis, and vitamin digestion and absorption in the infarcted area, enhance oxidative phosphorylation and NAD^+^ biosynthesis pathways, and ultimately improve myocardial energy metabolism and reducing fibrosis. Additionally, the PSTM hydrogel can solve the survival and implantation problems of ADSC in the infarction area. This pH-responsive, conductive hydrogel mimics the electrical mechanical environment of natural myocardium and can control the release of MXene-TA. MXene-TA regulates NAD^+^ levels and promotes the binding of NAD^+^ and CX43, thereby increasing the synthesis and release of SDF-1, significantly prolonging the survival time of ADSC and supporting its ability to repair MI tissues and prevent fibrosis. The conductive property of the hydrogel enhances the electrical coupling between myocardial cells, ultimately promoting long-term functional cardiac improvement. Compared with previously reported cardiac patches or injectable materials, our hydrogel system uniquely combines metabolic reprogramming and enhanced electrical conduction, addressing the 2 main obstacles in the MI regeneration process: redox imbalance and cell integration problems.

Our research shows MXene-TA has promising organ-selective anti-aging effects and improves cardiac function post-MI in rats. Limitations include unvalidated applicability to larger animals/humans due to species-specific metabolic/physiological differences and the need to evaluate long-term biodegradability (critical for chronic use) and immune responses (e.g., chronic inflammation from accumulation). These gaps outline a translational roadmap: large-animal studies (e.g., pigs) to validate efficacy/safety, biodegradation kinetic assessments, and immune profiling. With these steps, MXene-TA could emerge as a multifunctional therapy, applicable to (a) acute MI adjunct treatment (via NAD^+^ regulation and ischemia–reperfusion injury reduction), (b) stem cell implantation support (optimizing graft microenvironments through antioxidant/NAD^+^ enhancement), and (c) systemic aging management (targeting multi-organ dysfunction via organ-selective activity). Rigorous validation would position it as a novel intervention for age-related organ decline.

In conclusion, our study identifies NOX-mimetic nanozymes as a new therapeutic approach for both aging and MI, by catalytically restoring redox homeostasis, alleviating cellular senescence, and promoting stem cell-based repair. The integration of MXene-TA into a biofunctional hydrogel platform not only improves ADSC survival but also creates a supportive microenvironment for myocardial repair. This work provides a foundational basis for future translational development of redox-regulating biomaterials in cardiovascular medicine and aging-related tissue degeneration.

## Materials and Methods

### Animals and reagents

Male Sprague–Dawley rats (7 to 8 weeks old, 220 ± 20 g) were provided by the Laboratory Animal Center of Southern Medical University. All procedures involving animals were performed in compliance with institutional ethical guidelines and were approved by the Southern Medical University Animal Ethics Committee (SYXK[yue]2021-0167).

PVA (99.0%, molecular weight 89,000 to 98,000) was purchased from Sigma-Aldrich. Sodium alginate (viscosity 470 mPas at 20 °C) was obtained from Qingdao Hyzlin Biology Development Co. Ltd., and sodium tetraborate (analytical grade, borax) was obtained from Sinopharm Co. Ltd. Alexa Fluor 568-conjugated donkey anti-rabbit immunoglobulin G (IgG) (H&L) and Alexa Fluor 488-conjugated donkey anti-mouse IgG (H&L) were supplied by Invitrogen (USA). The Cell Counting Kit-8 (CCK-8) was obtained from New Cell and Molecular Biotech (China). Fluo-4 AM and WGA reagents were purchased from AAT Bioquest (USA). Primary antibodies targeting CX43 [ab11370, Western blot (WB) use concentration:1 μg/ml, immunofluorescence (IF) use concentration: 2 μg/ml], sarcomeric α-actinin (ab9465, IF use concentration: 2 μg/ml), CREB (phospho-S133) (P-CREB, ab32096, WB use concentration: 1 μg/ml), cAMP response element protein (CREB, ab32515, WB use concentration: 1 μg/ml), Sp1 transcription factor (SP-1, ab231778, WB use concentration: 1 μg/ml), C-X-C motif chemokine ligand 12 (SDF-1, ab155090, WB use concentration: 1 μg/ml), α-SMA (ab244818, IF use concentration: 1 μg/ml), vWF (ab6994, IF use concentration: 1 μg/ml), mannose receptor (CD206, ab64693, IF use concentration: 2 μg/ml), and CD86 (ab220188, WB use concentration: 1 μg/ml) were purchased from Abcam (Cambridge, UK).

### Synthesis of MXene materials

Six transition metal-based MXene materials (Ti₃C₂, Mo₂C, VOx-V₂C, Nb₄C₃, Ti₃C₂/Gu, and Ti₃C₂/Fe) were synthesized via a modified chemical exfoliation method. VOx-V_2_C MXene was prepared by hydrothermal treatment of V₂O₅ precursor with layered V₂AlC MAX phase at 180 °C for 12 h, autoclave pressure: ~1.2 MPa, followed by acid stripping using 1 M HCl/H₂O solution. Transition metal (Gu or Fe) doping into Ti₃C₂ was achieved through in situ reduction of metal salts (e.g., GuCl₃·6H₂O or FeCl₃·6H₂O) during the exfoliation process. All materials were washed thoroughly with deionized water and dried at 60 °C for 24 h. Tannic acid (0.1 mg/ml) was added to VOx-V_2_C solutions (10 μg/ml), stirring at 25 °C for 6 h to form vanadium–tannin complexes via chelation.

### Hydrogel fabrication

A pH-responsive conductive hydrogel (PSTM) was synthesized by cross-linking PVA (molecular weight = 150,000), OSA, borax, and tannic acid. OSA was prepared via oxidation of sodium alginate (NaAlg) with NaIO₄ (0.6 M) in ice-cold H₂O for 3 h. The PVA–OSA mixture (3:1, w/w) was then neutralized with NaOH (0.1 M) to form a homogeneous solution. Borax (0.5%, w/v) was added to introduce borate ester bonds for pH responsiveness, while tannic acid (0.5%, w/v) was incorporated to enhance self-adhesion. MXene-TA (0.2 to 1%, w/v) was dispersed into the pre-gel solution via sonication (30 kHz, 5 min) and mixed thoroughly. The final hydrogel was formed by freeze–thaw cycling (−80 °C/room temperature) for one cycle.

### Characterization of material

#### Structural analysis

AFM and SEM: Surface morphology and layer spacing of material were analyzed using AFM (Bruker Dimension Icon) and SEM (FEI Quanta 650), respectively. Samples were sonicated in ethanol (30 kHz, 1 min) and deposited onto silicon wafers.

DLS: DLS (Malvern Zetasizer Nano S) was used to measure the hydrodynamic diameter of MXene-TA after ultrasonication (30 kHz, 5 min).

EDS mapping: EDS was performed to analyze elemental distribution. The top 3 elements in PSTM were confirmed as C, O, and V, with vanadium uniformly dispersed throughout the scaffold.

XPS: XPS (Thermo Scientific Nexsa) was used to analyze the surface structure of MXene-TA, while electron paramagnetic resonance (Bruker E500-9.5/12) was employed to evaluate surface vacancies.

#### pH responsiveness

The pH-dependent release of MXene-TA from PSTM was evaluated by incubating hydrogels in NADH solutions (pH 6.5, 7.4, 8.8) for 20 h. NADH conversion rates were quantified colorimetrically using WST-8 (Dojindo) at 450 nm.

#### Electrical conductivity

CV was performed using a CHI 760E potentiostat. Electrodes were glassy carbon, and the electrolyte was 0.1 M KCl. The conductivity of PST and PSTM hydrogels was calculated based on the slope of the linear region of CV curves.

#### Mechanical properties

Elastic modulus: The elastic modulus of hydrogel samples was measured using a universal testing machine (Instron 5567) under compression at a crosshead speed of 1 mm/min until 50% strain. The modulus was calculated from the initial linear region of the stress–strain curve. Fatigue resistance: Cyclic compression tests (50% strain, 3,000 cycles) were conducted to evaluate stress retention.

#### Cytotoxicity assay

Cardiomyocytes were maintained in Dulbecco’s modified Eagle’s medium (DMEM) containing 15% fetal bovine serum (FBS). For cytotoxicity assessment, cells were exposed to VOx-V_2_C MXene (0 to 80 μg/ml) for 24 h, and viability was evaluated using the CCK-8 and LIVE/DEAD assays (Dojindo).

#### NOX activity assay (colorimetric detection)

The NADH oxidation activity of MXene materials was determined using the WST-8 colorimetric substrate. Reactions were performed in 96-well plates containing 100 μl of NADH solution (1 mM) and varying concentrations of MXene (0 to 100 μg/ml) at 37 °C. After 2 h, absorbance at 450 nm was measured using a microplate reader (SpectraMax i3).

#### Long-term NADH clearance and recycling

NADH clearance: MXene-TA (0 to 100 μg/ml) was incubated with NADH (1 mM) for 15 min. The remaining NADH concentration was quantified using an ultraviolet–visible (UV-Vis) spectrophotometer (λ = 340 nm).

NAD^+^/NADH cycle: After 24 h of MXene-TA treatment, GDH (1 U/ml) was added to convert NAD back to NADH. Changes in absorbance at 340 nm were monitored for 30 min.

#### Cell culture and treatment

Primary neonatal rat ventricular myocytes were isolated from 1- to 3-day-old Sprague–Dawley rats using previously described protocols [40,41,50]. Cardiac tissues were digested with 0.25% trypsin and 0.1% collagenase type II to obtain single-cell suspensions. The cells were centrifuged at 900*g* for 5 min and pre-plated for 2 h to reduce nonmyocyte contamination.

Rat white adipose-derived ADSCs were cultured in DMEM supplemented with 15% FBS. For hydrogel seeding, ADSCs (2 × 10^5^ cells/ml) were mixed with PSTM hydrogel precursor and cultured at 37 °C in a humidified atmosphere.

#### Cellular uptake and catalytic activity analysis

### Cellular uptake analysis

MXene-TA nanoparticles were conjugated with rhodamine B isothiocyanate (RBITC) via amide coupling. ADSCs were seeded on glass coverslips and treated with RBITC-labeled MXene-TA (4 μg/ml) for 24 h. Cell membranes were counterstained with WGA (Alexa Fluor 488, 1 μg/ml) for 15 min. Confocal microscopy (Zeiss LSM880) was used to visualize nanoparticle localization, with images captured at 63× magnification.

#### NAD^+^/NADH ratio quantification

ADSCs were treated with MXene-TA (2 to 8 μg/ml) for 24 h. Intracellular NAD^+^ and NADH levels were quantified using a commercial kit (Abcam, catalog no. ab197223). The NAD^+^/NADH ratio was calculated as the quotient of absorbance at 340 nm (NADH) and 630 nm (NAD^+^).

#### Senescence induction and marker analysis

ADSCs were induced to senescence by treatment with d-galactose (100 mM) for 7 d. SA-β-gal activity was detected by X-galactosidase staining (5 mM, pH 6.0) for 16 h. Telomere length was measured by quantitative real-time polymerase chain reaction (qRT-PCR) using primers for telomerase reverse transcriptase (hTERT) and glyceraldehyde-3-phosphate dehydrogenase (GAPDH). The expression of p16 (Cdkn2a) and p21 (Cdkn1a) was quantified by qRT-PCR with SYBR Green Master Mix.

#### Cell proliferation and viability

ADSCs were seeded onto hydrogels containing 0.2%, 0.4%, 0.6%, or 1% MXene-TA. Cell viability was evaluated using the Live/Dead assay (Dojindo) after 72 h. Proliferation was assessed via EdU incorporation (Invitrogen) after 48 h. Images were acquired with confocal microscopy (Zeiss LSM 880).

#### ROS and mitochondrial membrane potential

Primary cardiomyocytes were treated with MXene-TA (2 to 8 μg/ml) for 24 h. ROS levels were quantified using 2′,7′-dichlorodihydrofluorescein diacetate (DCFH-DA). Mitochondrial membrane potential was assessed using JC-1 dye (5 μM), with green (low potential) and red (high potential). Images were acquired with a fluorescence microscope (Olympus BX53 software).

#### Cytokine secretion analysis

ADSCs were cultured on PSTM hydrogels containing 0% to 1% MXene-TA. After 72 h, the conditioned medium was collected, and the levels of 13 cytokines—SDF-1, SCF-1, IGF-1, TGF-β, EGF, VEGF, LIF, M-CSF, IL-6, Ang-1, HGF, FGF, and PDGF-AA—were measured using commercially available ELISA kits from eBioscience (San Diego, CA, USA).

#### SPR analysis

The binding affinity of NAD^+^ to CX43 was determined using a Biacore T200 system. Recombinant human CX43 (GenScript) was immobilized on a CM5 sensor chip. NAD^+^ solutions (0 to 1,000 μM) were injected, and dissociation constants (*K*_D_) were calculated.

#### Protein modeling and molecular docking

The CX43 protein structure was modeled using the AlphaFold server and optimized with Discovery Studio. The initial structure of the NAD^+^ molecule was obtained from ZINC15. Autodock Vina performed the molecular docking of CX43 to NAD^+^, and the highest-scoring complex structure was selected for further analysis. The docking results were visualized using Pymol and Discovery Studio.

#### Western blot analysis

Cell-derived protein samples were separated by sodium dodecyl sulfate–polyacrylamide gel electrophoresis (SDS-PAGE) and transferred onto polyvinylidene difluoride (PVDF) membranes (Millipore, Billerica, MA, USA). Membranes were blocked with tris-buffered saline (TBS) containing 0.05% Tween 20 and 5% bovine serum albumin (BSA) for 1 h at room temperature, followed by incubation with primary antibodies either overnight at 4 °C or for 2 h at room temperature. After washing, membranes were incubated with horseradish peroxidase (HRP)-conjugated secondary antibodies for 1 h at room temperature. Protein signals were then visualized using enhanced chemiluminescence (Thermo Fisher, Carlsbad, CA, USA) according to the manufacturer’s instructions.

#### Immunostaining

Immunofluorescence staining was performed on rabbit anti-CX43, mouse α-actinin, mouse anti-α-SMA, rabbit anti-vWF, WGA-FITC (fluorescein isothiocyanate) (dilution 1:200), CD86, and CD206 at 4 °C overnight. Subsequently, they were treated with Alexa Fluor 488 or Alexa Fluor 568 fluorescent secondary antibody (diluted to 1:500) for 1 h. Subsequently, the stained sample was incubated with 4′,6-diamidino-2-phenylindole (DAPI) for 10 min to observe the nucleus. A fluorescence microscope (Olympus BX53 software) was employed to acquire the images.

#### Establishment of MI model and hydrogel implantation

Male Sprague–Dawley rats (7 to 8 weeks old, 220 ± 20 g) were anesthetized with isoflurane (3% to 4% induction, 1.5% to 2.5% maintenance) and mechanically ventilated. Following a left lateral thoracotomy and pericardiectomy, the LAD coronary artery was ligated to induce MI. Animals were randomly assigned to 6 groups: Sham, MI, PST hydrogel, PST/ADSC, PSTM hydrogel, and PSTM/ADSC. Hydrogels (100 mg) with or without ADSCs (1 × 10^6^ cells/gel) were implanted into the infarcted region immediately after MI. Animals were euthanized 4 weeks post-surgery for subsequent analyses.

#### Measurement of cardiac function by echocardiography

Left ventricular function was evaluated using an IE33 echocardiography system (Vevo2100, Visual Sonics) at multiple time points following MI induction. Rats were anesthetized with isoflurane (3% induction, 2% maintenance). M-mode and B-mode images of the anterior left ventricular wall and beat-to-beat activity were acquired with an M250 probe. Cardiac function parameters, including fractional shortening (FS) and ejection fraction (EF), were calculated from 3 consecutive cardiac cycles.

#### Histological assessment

Rats were euthanized at designated time points, and hearts were collected for analysis. Frozen sections (6 μm) were prepared and subjected to Masson’s trichrome staining to evaluate histological features of MI. Infarct size was quantified by calculating the ratio of collagen (blue) to myocardial tissue (red) using the stained sections. For assessment of tissue damage, paraffin-embedded heart sections (5 μm) were mounted on polylysine-coated slides and stained with H&E. Staining results were analyzed using ImageJ software.

#### Epicardial activation mapping

Epicardial electrical propagation between healthy and infarcted myocardium was assessed using a 64-channel electrode array mapping system (MappingLab, Oxfordshire, UK). At 28 d post-hydrogel implantation, rats were euthanized, and hearts were rapidly excised and perfused via a Langendorff system. A 64-electrode array was positioned at the border zone between healthy and infarcted tissue to record spontaneous local field potentials. A stimulus electrode was placed on the epicardium beneath the left atrial appendage to deliver electrical pulses (4 V, 5 Hz) for mapping local field potentials and activation times. Conduction velocity and activation patterns were analyzed using EMapScope software, and heat maps of electrical conduction were generated.

### Statistical analysis

Data were analyzed using SPSS 22.0. Normality of the data was assessed with the Shapiro–Wilk or Kolmogorov–Smirnov test. Data with a normal distribution are presented as mean ± SD. Comparisons between 2 groups were performed using an unpaired 2-tailed Student’s *t* test, while multiple group comparisons were conducted by one-way analysis of variance (ANOVA) followed by Bonferroni’s post hoc test. Statistical significance was defined as *P* < 0.05.

## Data Availability

All data needed to evaluate the conclusions in the paper are present in the paper and/or the Supplementary Materials. Additional data related to this paper may be requested from the authors. Source data are provided in this paper.
