# Supplementary material for: A NADH Oxidase Nanozyme Restores Redox Homeostasis to Ameliorate Multi-Organ Aging and Ischemic Cardiomyopathy
Source: Research (Wash D C). 2025 Oct 28;8:0973. doi: 10.34133/research.0973 (PMC12559800; doi:10.34133/research.0973)
Supplement: Supplementary 1 — Figs. S1 to S9 Movie S1 [file research.0973.f1.zip › supplementary materials.docx]

Supplementary Materials for

**A NADH-Oxidase Nanozyme Restores Redox Homeostasis to Ameliorate Multi-Organ Aging and Ischemic Cardiomyopathy**

Yu Chen *et al.*

*Corresponding author. Email: [qqiuxzh@163.com,](mailto:qqiuxzh@163.com,) ss.hhh89@hotmail.com.

**This PDF file includes:**

Figs. S1 to S8

Fig. S1.


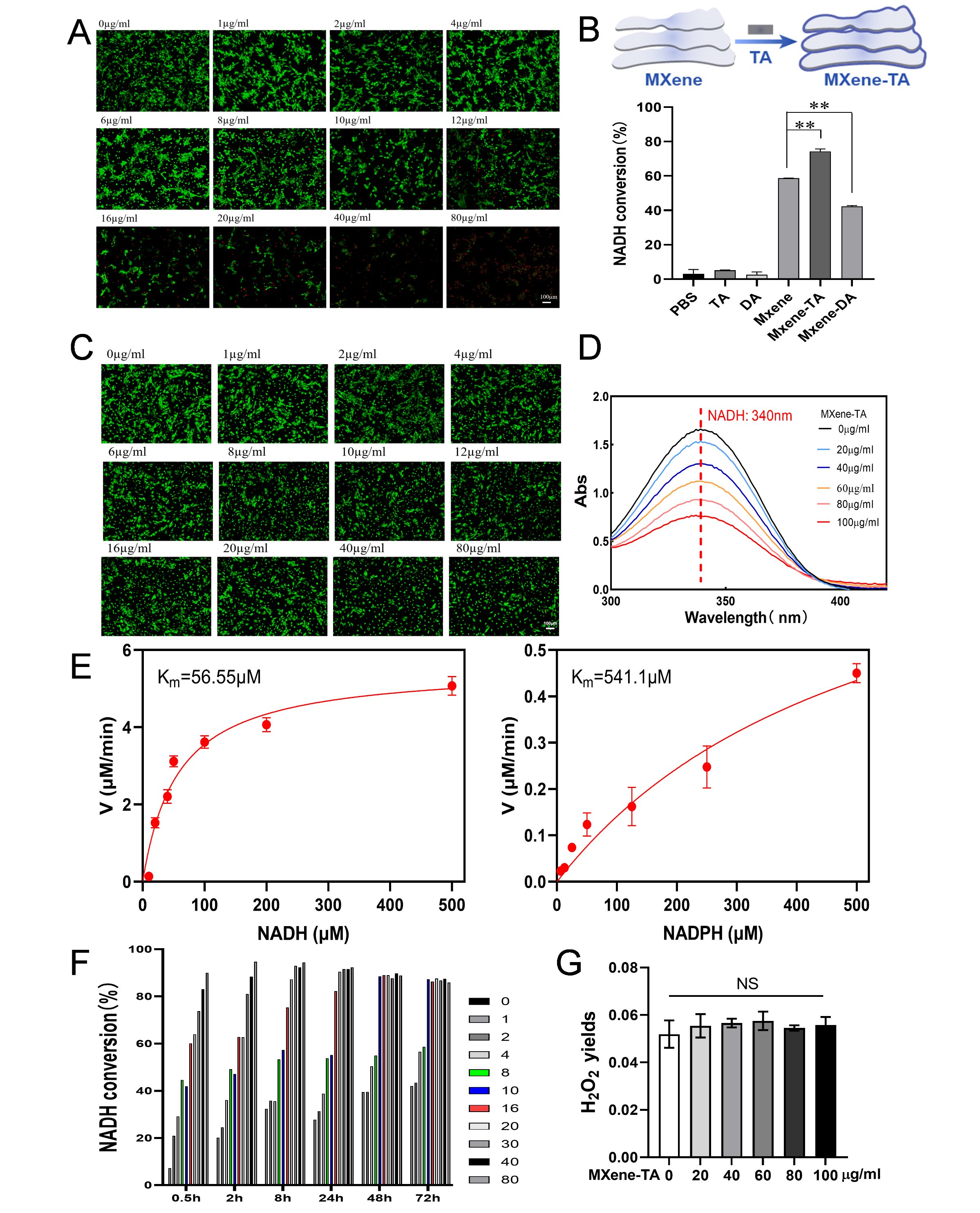


**Fig.S1 Detection of MXene material biocompatibility and NADH oxidase activity. (A).** After the primary cardiomyocytes were treated with different concentrations of MXene (0-80μg/ml) for 24 hours, the toxicity of the materials was detected by live staining, Bar=100μm. **(B).** Schematic diagram of MXene-TA synthesis process, and assessment of catalytic activities of MXene materials by the absorbance changes of the substrate in the WST-8 assay, n=3.**(C).** Cardiomyocytes were treated with different concentrations of MXene-TA (0-80ug/ml) for 24 h, and the living and dead cells were stained, Bar=100μm. **(D).** Assessment of catalytic activities of MXene-TA by the Ultraviolet spectrum. **(E).** Kinetic analysis showed that the Km of MXene-TA for the substrate NADH and NADPH. **(F).** Different concentrations of MXene-TA (0-80ug/ml) were added to the NADH solution, and the NADH conversion rate was detected within 72 hours, n=3. **(G).** The amount of hydrogen peroxide produced during the catalytic NADH process by MXene-TA for 24 h was detected using a colorimetric assay. All data are presented as mean ± SD. *P < 0.05, **P < 0.01.

Fig. S2.


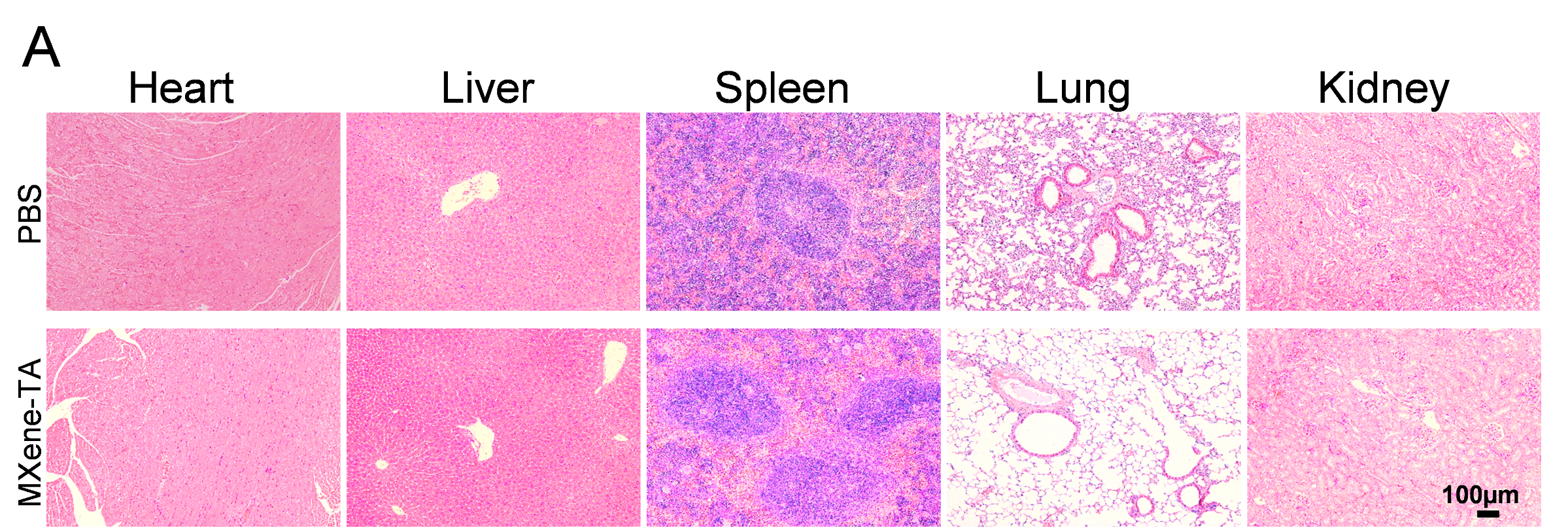


**Fig.S2 Safety of MXene-TA. (A).** 24-month-old naturally senescent C57BL/6 mice were selected and randomly grouped and then treated with daily intraperitoneal injections of PBS or MXene-TA (8 µg/mL) for six consecutive weeks, H&E staining of different organs, n=5, Bar=100μm.

Fig. S3.


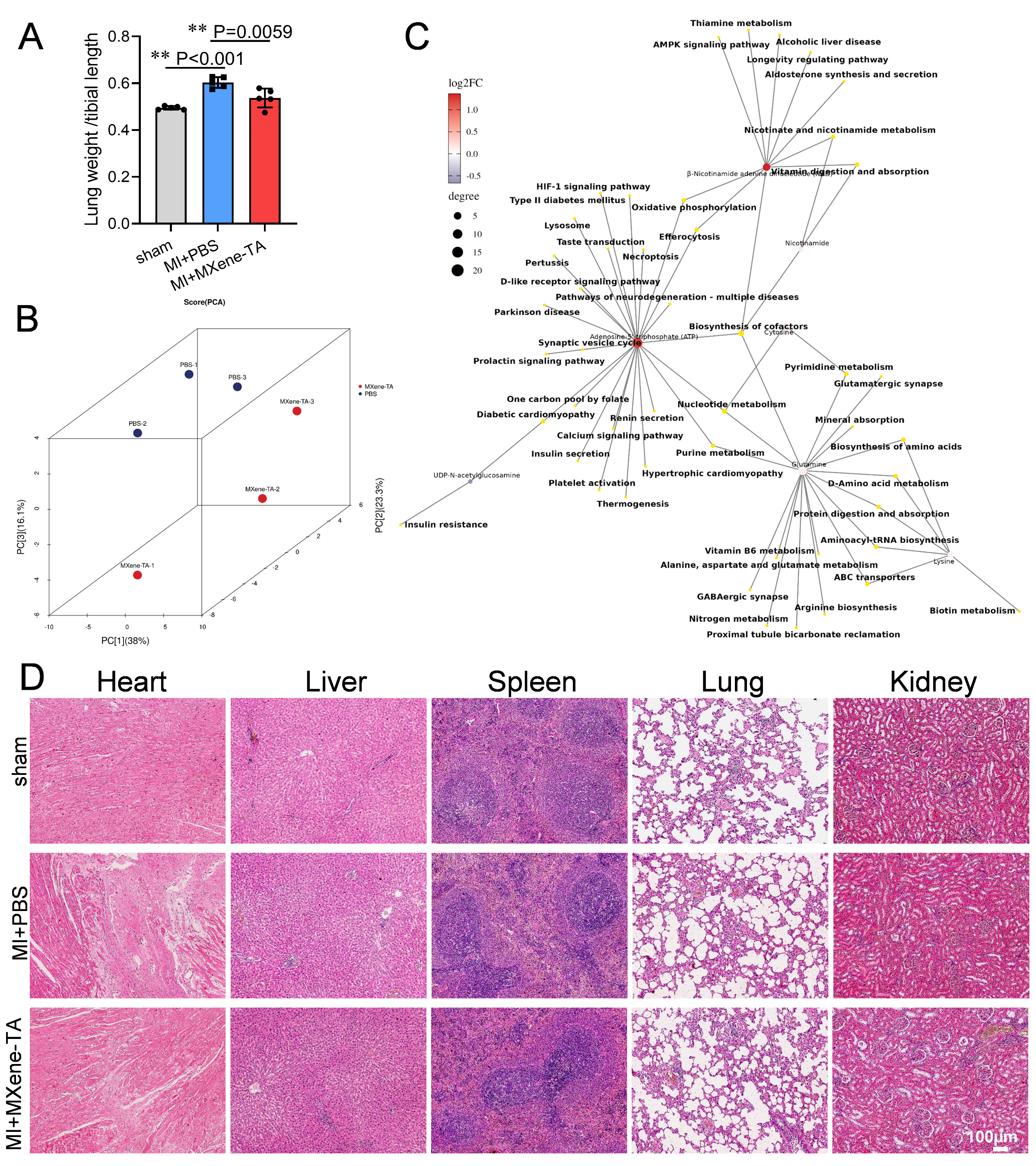


**Fig.S3 Safety and efficacy of MXene-TA.** MI was induced via permanent ligation of the left anterior descending (LAD) coronary artery, followed by a single injection of either MXene-TA (8 μg/mL) or PBS into the infarct border zone. **(A).**Lung weight/tibia length (LW/TL) ratios was detected after 4 weeks. **(B).** PCA analysis of the PBS group and the mxene-TA group. **(C).** KEGG pathway diagram. **(D).** H&E staining of different organs, n=5, Bar=100μm.

Fig. S4.


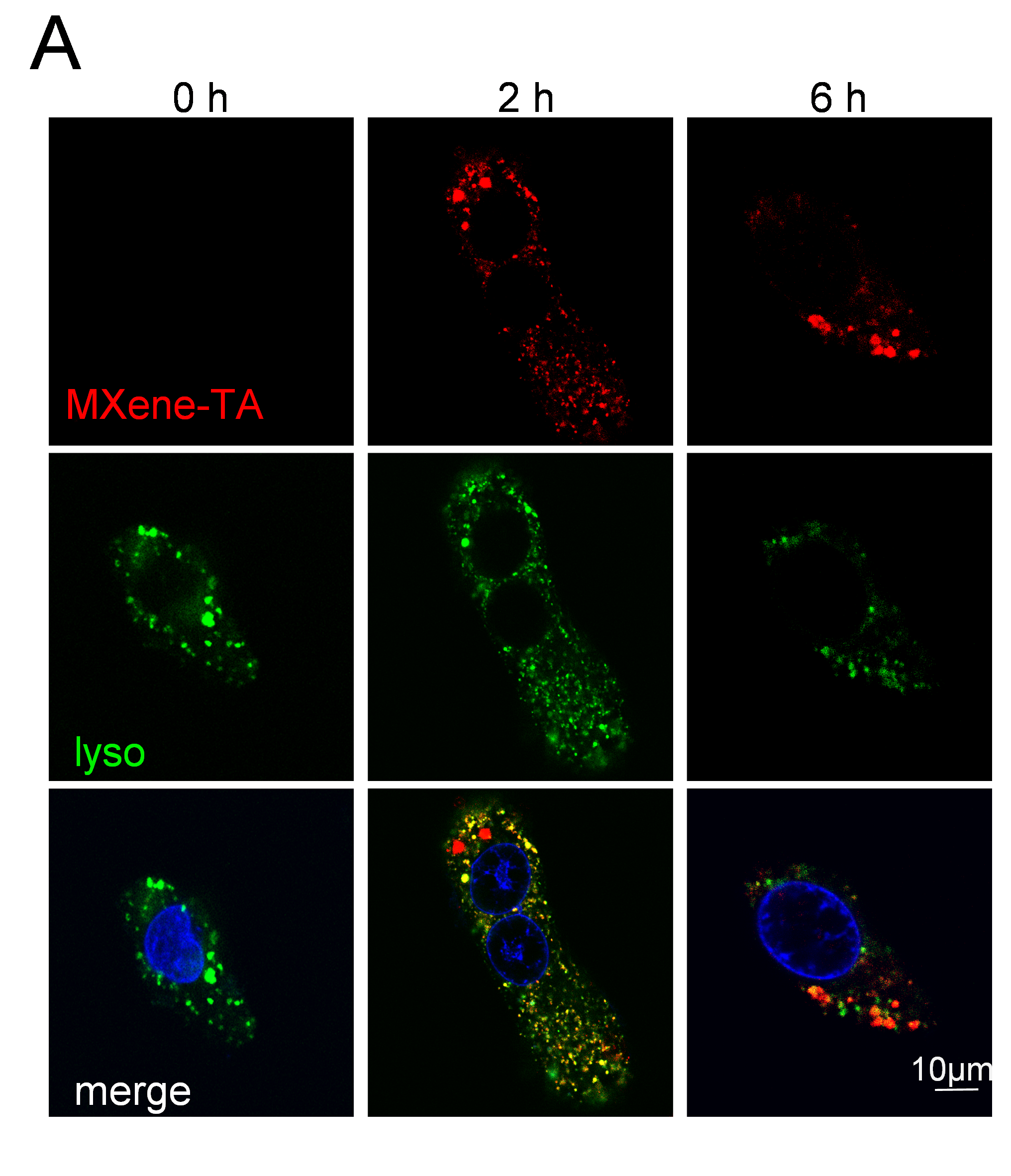


**Fig.S4** Fluorescent confocal laser scanning microscopic images of ADSC after incubation with MXene-TA within 6h, red fluorescent images of Rhodamine B-labeled MXene-TA; green fluorescent images of endolysosomes stained by Lysotracker; bule fluorescent images of the nucleus, Bar= 10 μm.

Fig.S5.
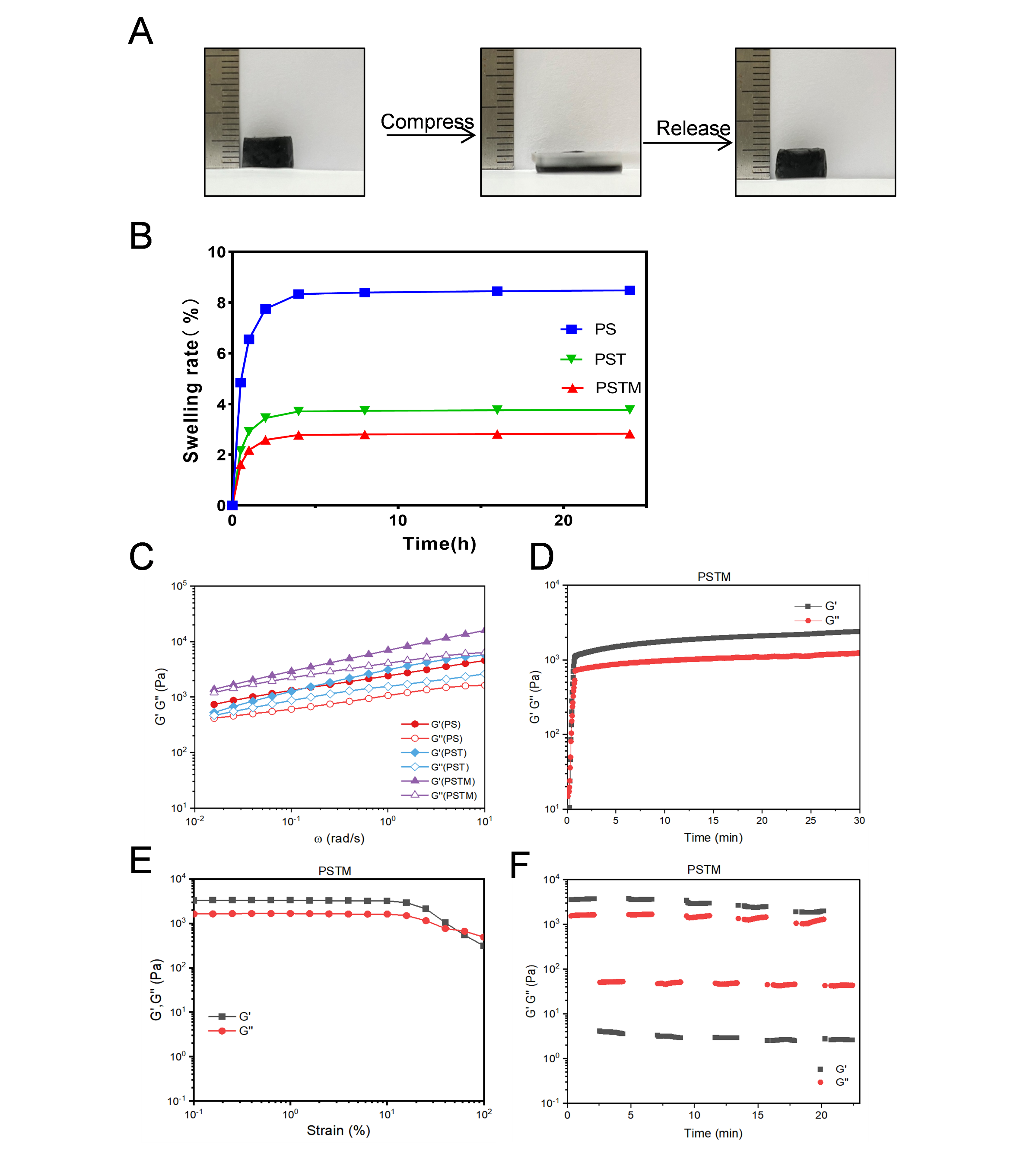


**Fig.S5 Morphological characteristics of hydrogels.** **(A).** Physical image of PSTM hydrogel after compression and recovery. **(B).** Within 24 hours, the swelling rate of different hydrogels was detected, n=3. **(C-F).** Detection of the rheological properties of hydrogels.

**Fig.S6**

**
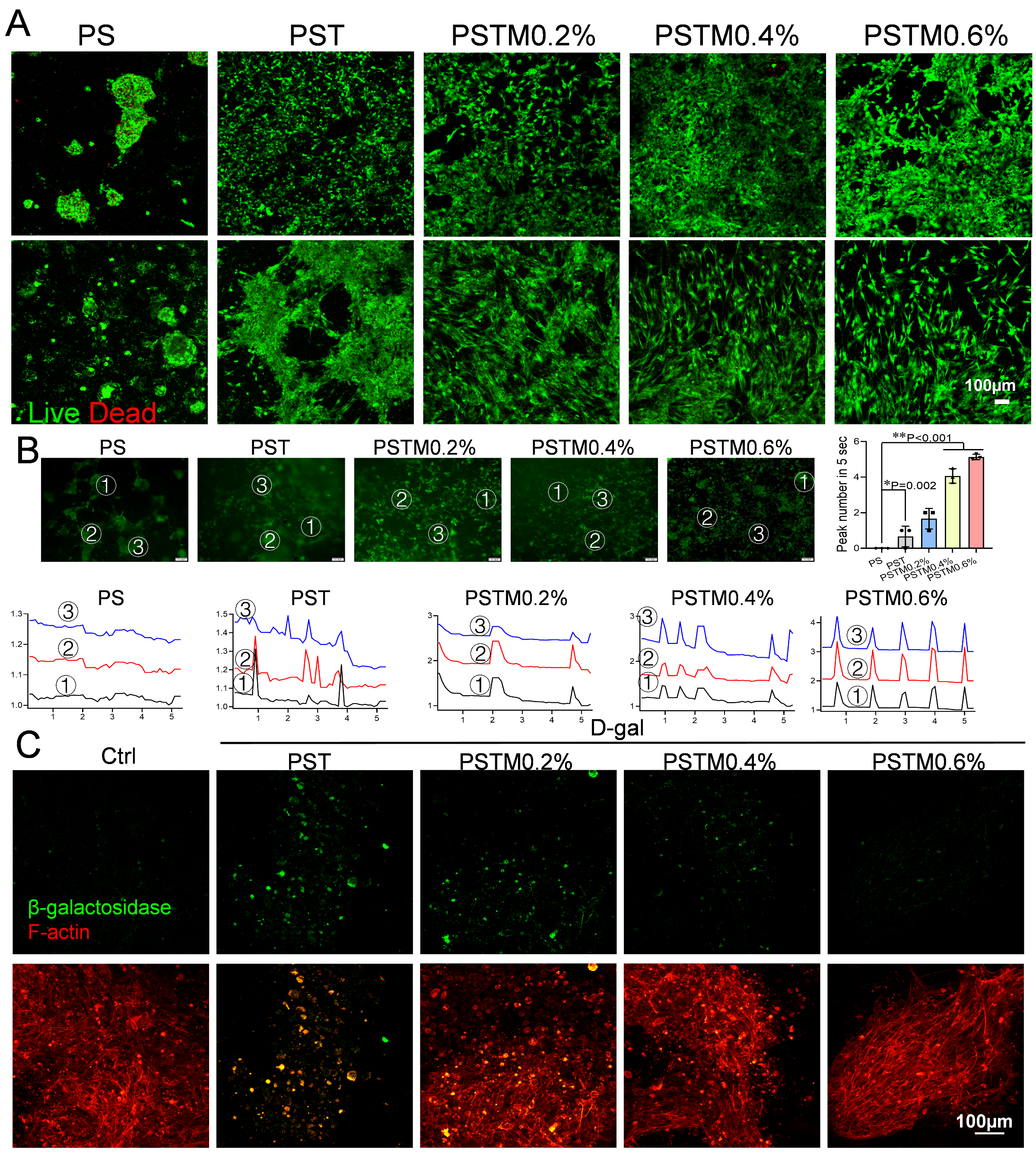
**

**Fig.S6 The biocompatibility, conductivity and biological activity of PSTM hydrogel. (A).** Cardiomyocytes(up panel) and ADSCs(down panel) were implanted on different hydrogels for 72 hours, and the biocompatibility of the hydrogels was detected with LIVE and DEAD staining, n=3, Bar=100μm. **(B).** Calcium transient (up) and corresponding Ca2+ frequency signals (bottom) in CMs on different scaffolds at day 7, n=3. **(C).** ADSCs were planted on culture dishes or different hydrogels. After D-galactose induced cell senescence, β-galactosidase staining was used to detect cell senescence level, n=3, Bar=100μm. Quantitative data were expressed as the mean ± SD. of at least 3 independent experiments. *P < 0.05, **P < 0.01.

Fig. S7.

**
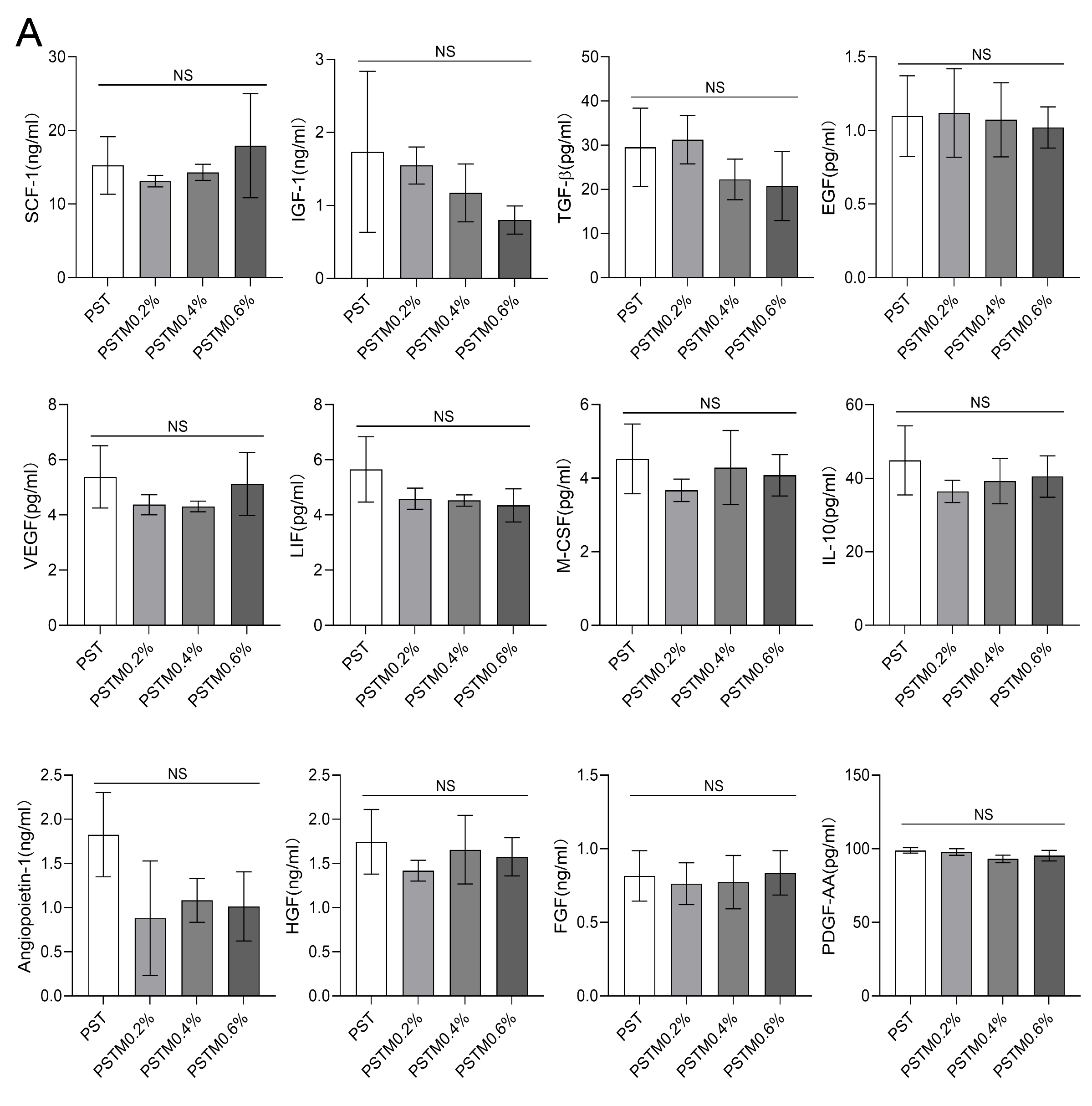
**

**Fig.S7 Detection of cytokines secreted by mesenchymal stem cells on hydrogel. (A).** ADSCs were cultured on different hydrogels for 3 days, the supernatants were collected, and the Elisa assay was used to detect the expression of cytokines, n=3 rats. All data are presented as mean ± SD. NS, not significant.

Fig. S8.


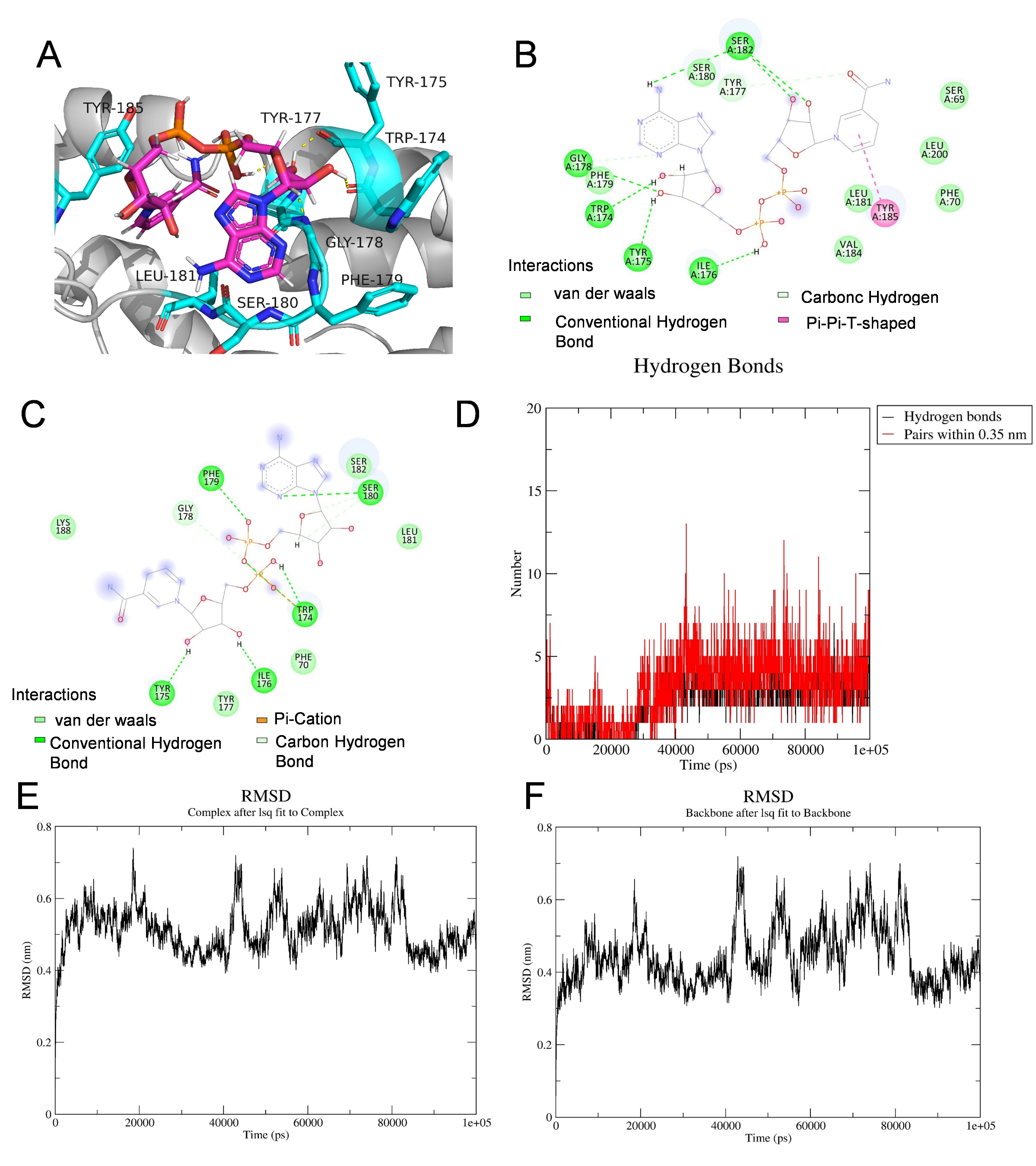


**Fig.S8 NAD^+^ bind to CX43. (A).** Molecular docking mimics the binding mode and binding site of NAD^+^ to of CX43. **(B).** Molecular docking predicts the interaction force and binding site of NAD^+^ with CX43. **(C).** Molecular dynamics predicts the interaction force and binding site of NAD^+^ with CX43. **(D).** Molecular dynamics statistics on the number of hydrogen bonds of CX43 protein interacting with NAD^+^. Molecular dynamics statistics root mean square deviation (RMSD) of CX43 protein in complex with NAD^+^**(E)** and CX43 protein**(F)**.

Fig. S9.


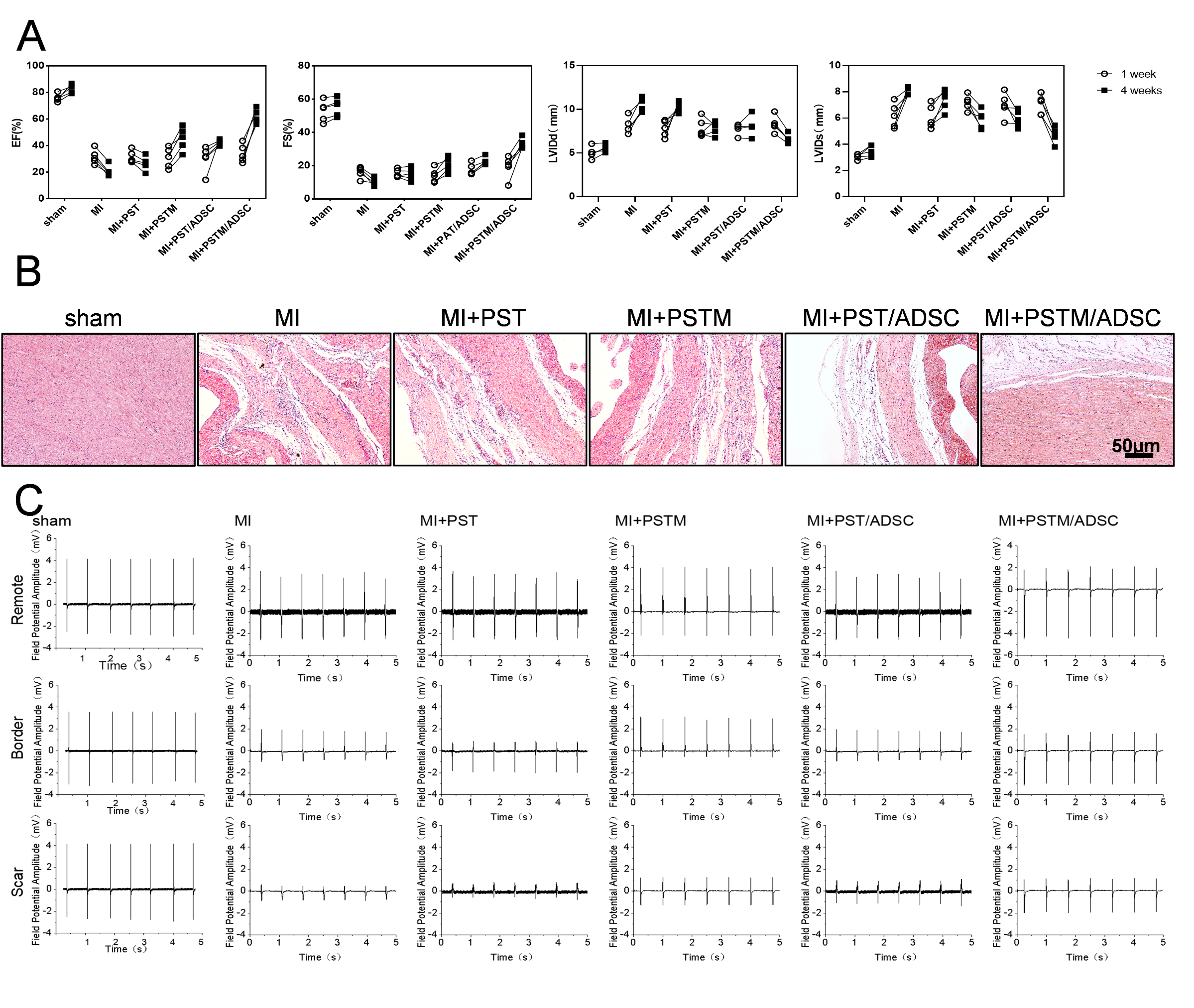


**Fig.S9 PSTM/ADSC hydrogel repaired myocardial infarction. (A).** In different groups, FS, EF, LVIDs, and LVIDd were determined by echocardiography on day 7 and day 28 after implantation, n=5. **(B).** Different hydrogels implanted in infarcted rats and H&E staining of infarcted areas after 28 days, n=5, Bar=50μm. **(C).** Without additional stimuli, a 64-channel electrophysiologic calibration system was used to detect the amplitude of spontaneous potentials in different groups of noninfarcted, bordered, and infarcted areas, n=5.
